# Dermal Exposure to Bisphenols and Their Analogs: A Systematic Review and Narrative Synthesis

**DOI:** 10.1007/s40572-026-00574-2

**Published:** 2026-11-13

**Authors:** Joseph Ondari Nyakundi, Jun Wang

**Affiliations:** Division of Environmental and Industrial Hygiene, Department of Environmental and Public Health Sciences, University of Cincinnati, 160 Panzeca Way, Cincinnati, OH 45267-0056 USA

**Keywords:** Bisphenols, Dermal exposure, Skin absorption, Thermal paper, Toxicokinetics, PBPK modeling

## Abstract

**Purpose of Review:**

Dermal exposure to bisphenols is an underrecognized pathway of systemic uptake, but differences in study designs and measured endpoints complicate exposure assessment. This systematic review synthesizes 71 studies examining dermal transfer, skin permeation and retention, human toxicokinetics, and exposure modeling for bisphenol A (BPA) and its analogs.

**Recent Findings:**

BPA has the strongest evidence for systemic dermal uptake, supported by controlled human and experimental studies, while occupational biomonitoring supports exposure relevance without isolating the dermal route. Human studies demonstrate delayed BPA absorption and partial avoidance of first-pass metabolism. BPS generally shows lower transdermal passage than BPA but substantial skin retention, whereas BPAF and TGSA exhibit appreciable permeation in human-skin experiments. Thermal paper remains the best-characterized source; textiles, hygiene products, and other consumer materials represent additional potential sources, although their dermal dose estimates rely largely on modeled migration and absorption assumptions.

**Summary:**

Dermal exposure assessment requires compound- and condition-specific parameters that distinguish skin loading, retention, permeation, and systemic bioavailability. Lower permeability does not necessarily imply lower risk. Improved human toxicokinetic data, realistic migration measurements, and refined PBPK models are needed to strengthen exposure assessment and evaluation of bisphenol alternatives.

**Supplementary Information:**

The online version contains supplementary material available at https://doi.org/10.1007/s40572-026-00574-2.

## Introduction

Bisphenol A (BPA) and related compounds occur in plastics, resins, thermal paper, textiles, and other consumer materials, resulting in widespread human exposure through multiple pathways [1–8]. ​BPA​ remains the most extensively studied compound in this class. Substitution has increased interest in bisphenol S (BPS), bisphenol F (BPF), bisphenol AF (BPAF), and alternative thermal-paper developers following regulatory pressure and market demand for “BPA-free” materials. However, selected substitutes have shown biological activity in experimental systems highlighting that neither structural similarity to BPA nor lower skin permeability establishes comparative safety [3, 6, 8].

Dietary intake is an important source of bisphenol exposure through migration from food-contact materials, while inhalation and dermal contact provide additional exposure pathways, particularly in indoor and occupational environments [1, 9–13]. These concerns have informed regulatory action, including the European Union’s restriction of BPA in thermal paper [14, 15]. Growing evidence suggests that dermal exposure may represent an important yet underrecognized route of systemic uptake, especially among individuals frequently handling thermal paper and other bisphenol-containing materials [16, 17]. Unlike oral ingestion, dermal absorption can partially bypass presystemic metabolism, potentially influencing systemic bioavailability and internal exposure [18–21]. However, the magnitude and relative contribution of this pathway remain insufficiently characterized. Occupational biomonitoring supports additional exposure; labeled controlled studies more directly address route-dependent kinetics and unconjugated parent fractions [22–25].

Evidence on dermal bisphenol exposure spans material-to-skin transfer studies, in vitro and ex vivo skin diffusion experiments, controlled human investigations, occupational biomonitoring, and exposure modeling [17, 22, 26–29].These approaches assess distinct aspects of exposure: product measurements identify potential sources, transfer studies characterize skin loading, diffusion experiments assess permeation and retention, and systemic biomarkers reflect uptake and disposition without necessarily distinguishing exposure routes [17, 22, 26–28, 30]. Differences in study designs, compounds, exposure conditions, and measured endpoints complicate cross-study comparisons and interpretation of dermal uptake, particularly for BPA substitutes, with implications for exposure assessment and physiologically based pharmacokinetic (PBPK) modeling.

This systematic review synthesizes evidence on skin transfer, permeation and retention, systemic uptake, and exposure modeling for BPA and its analogs. Through narrative synthesis and descriptive comparisons, it examines compound-specific differences, methodological variability, and implications for exposure assessment, PBPK modeling, and research priorities.

## Methods

Peer-reviewed original research articles published in English through March 2026 were identified through systematic searches of PubMed, Scopus, Web of Science, Embase, ScienceDirect, and SpringerLink. Searches were conducted from November 1, 2025, and completed on March 30, 2026, with search terms combining chemical identifiers (e.g., bisphenol, BPA, BPS, BPF, BPAF, BPB, TGSA, D-8, BPSIP) with dermal exposure concepts (e.g., dermal, skin, percutaneous, transdermal, permeation, skin absorption, Franz cell, OECD 428), source terms (e.g., thermal paper, receipt, cashier, textile, clothes, currency), and internal dose or modeling terms (e.g., urine, serum, plasma, biomonitoring, pharmacokinetics, toxicokinetics, PBPK, modeling). Multiple search-string combinations and variations in terminology were applied within each database to improve retrieval of studies addressing different aspects of dermal exposure. Search strings are provided in Supplemental Material S-1.

Studies were included if they investigated dermal exposure to BPA or related bisphenols and reported dermal-pathway outcomes such as transfer to skin, skin absorption or permeation, skin metabolism or retention, systemic biomarkers after dermal contact, or exposure/risk modeling based on explicit dermal inputs. Reviews, commentaries, abstracts, theses, non-peer-reviewed reports, and studies unrelated to skin contact were excluded. Regulatory and health-based guidance documents were used to contextualize exposure and risk interpretation but were not treated as included primary studies.

All retrieved records were consolidated in Zotero. Duplicates were removed before screening and extraction into a standardized spreadsheet. One reviewer screened titles, abstracts, and full texts, with decisions cross-checked by a second reviewer and disagreements resolved by consensus. Design-specific limitations were considered without a uniform formal risk-of-bias assessment. Heterogeneity precluded meta-analysis; evidence was synthesized narratively, with descriptive plots of receptor/transdermal recovery, skin retention, and permeability coefficients (Kp) for compatible human in vitro/ex vivo conditions. Conditions from the same study were not treated as independent, and no estimates were statistically pooled. Reporting followed PRISMA 2020 guidelines [31]. The study selection flow diagram was generated using the Shiny app [32], and the completed PRISMA checklist is provided in supplemental material S-2. The review protocol was not prospectively registered.

## Results and Discussion

### Overview of the Evidence Base

**Fig. 1:**
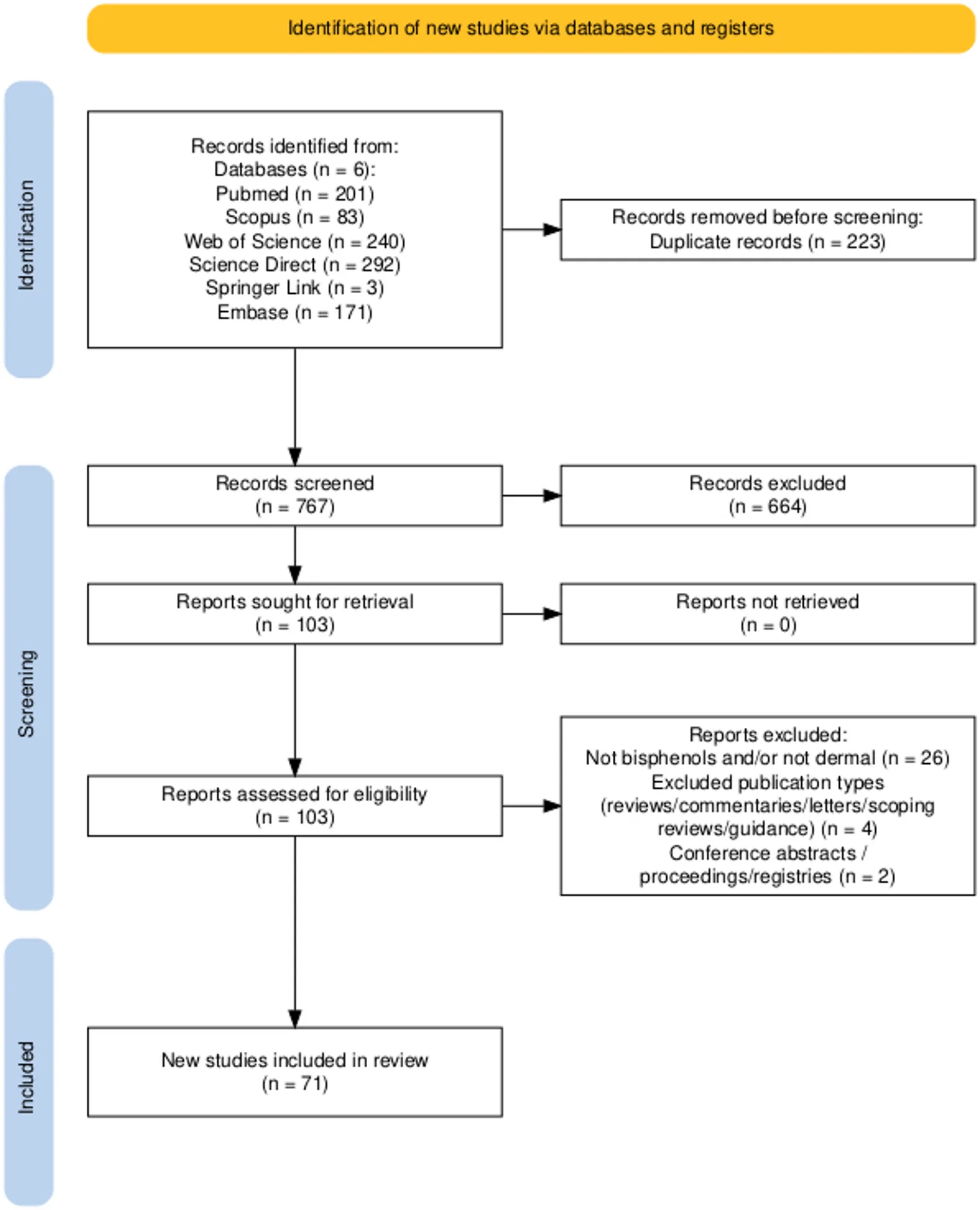
PRISMA flow diagram of the screening process

Database searches identified 990 records, of which 223 were removed as duplicates. Of the 767 records screened, 664 were excluded, and 103 full-text reports were assessed for eligibility. Following 32 full-text exclusions, 71 studies were included in the review (Fig. 1). The included literature spans experimental skin permeation, dermal transfer, human exposure investigations, occupational biomonitoring, and exposure modeling. While earlier research focused predominantly on BPA and thermal paper [17, 20, 26, 33–35], more recent investigations have expanded to bisphenol substitutes and other consumer-contact materials, although evidence remains uneven across compounds and exposure sources [27, 30, 36–45]. Detailed characteristics of the included studies and extracted findings are presented in supplemental tables S3–S9.

### In Vitro and Ex Vivo Studies

In vitro and ex vivo studies provide key mechanistic evidence of bisphenol skin permeation, retention, and cutaneous metabolism. Earlier investigations focused primarily on BPA [20, 33–35, 46], whereas later studies expanded to substitute bisphenols and systematically examined the effects of vehicle (water, acetone, artificial sebum)/occlusion [27, 30, 47], and model/species differences [46, 48, 49]. The studies revealed substantial model-dependent variability in dermal permeation and retention, with differences in skin preparation, barrier characteristics, and species influencing absorption estimates and limiting direct comparisons across experimental systems.

Direct comparative studies consistently demonstrate greater transdermal permeation of BPA than BPS, although the magnitude varies with experimental conditions. In reconstructed human epidermis, BPA exhibited higher permeability and receptor-fluid recovery than BPS [50], a pattern also observed in excised human skin across different vehicles [27]. However, BPA recovery varied considerably across experimental models. Demierre et al. (2012) reported 8.6% receptor recovery after 24 h [35], whereas Toner et al. (2018) observed 1.7–3.6% receptor recovery but 16–20% dermal delivery when radioactivity retained in the viable epidermis and dermis was included [51]. Earlier investigations also demonstrated substantial BPA permeation and retention under differing experimental conditions [20, 51]. Together, these findings indicate that apparent BPA absorption varies considerably with experimental conditions and measured endpoints, while appreciable skin retention may occur even when transdermal passage is limited.

BPS exhibits a distinct dermal disposition pattern characterized by lower transdermal passage than BPA but appreciable skin retention. In reconstructed human epidermis, BPS receptor recovery after 25 h was 6.4–8.0%, compared with 43–46% for BPA [50]. Similarly, in fresh human split-thickness skin, Champmartin et al. (2020) reported substantially lower BPS permeation across vehicles, with calculated 40-h receptor recovery of 0.055%, 1.28%, and 0.205% of the applied dose in acetone, artificial sebum, and water, respectively [27]. Despite this limited passage, 20–47% of the applied BPS dose remained within the skin, exceeding BPA retention under some matched conditions [27]. Thus, BPA and BPS differ not only in the magnitude of transdermal transfer, but also in how the dose partitions between receptor fluid and skin compartments. Hence, lower transdermal permeation does not necessarily indicate limited skin accumulation.

Beyond transdermal passage, experimental studies suggest that retained bisphenols may form cutaneous reservoirs capable of prolonging exposure after surface contact ends. For BPA, Demierre et al. (2012) recovered 34.9% of the applied dose in the stratum corneum after 24 h, compared with only 0.6% in the underlying skin membrane [35]. Other human skin studies similarly demonstrated appreciable BPA retention under differing experimental conditions [20, 33–35, 51]. For BPS, Marquet et al. (2024) found that substantial amounts remained in excised human skin, predominantly within the stratum corneum, even after surface washing, suggesting potential for continued diffusion following exposure [30]. Complementary rat studies demonstrated BPS persistence in skin beyond 72 h despite relatively low systemic absorption [36]. These observations indicate that dermal contact with bisphenols should not be evaluated solely during active contact; the skin may act as a temporary depot, potentially extending systemic input beyond the period of external contact, although the systemic availability of retained fractions remains uncertain.

Vehicle composition and occlusion emerged as important determinants of bisphenol dermal permeation, contributing to variability in absorption estimates. Champmartin et al. (2020) demonstrated substantial vehicle-dependent differences in BPA receptor recovery, ranging from 3% of the applied dose in artificial sebum to 41% in water after 40 h, with corresponding variation in skin retention [27]. Similarly, Marquet et al. (2024) reported that occlusion increased BPS absorption by up to six-fold, with effects varying by vehicle [30]. BPS, however, maintained very low receptor-fluid passage but substantial cutaneous retention across vehicles [27]. These findings highlight the importance of experimental conditions in determining dermal uptake and underscore the need to evaluate realistic exposure scenarios involving moisture, skin formulations, and occlusive contact.

Cutaneous metabolism of bisphenols was evident but varied across experimental models and skin compartments. Zalko et al. (2011) demonstrated BPA glucuronidation and sulfation in viable human and pig skin, with substantially greater metabolism in pig skin [20]. Similarly, Toner et al. (2018) detected BPA-glucuronide and BPA-sulfate in dermal tissue and receptor fluid, but not in epidermal samples, although metabolism was evaluated only at the highest tested concentration [51]. In contrast, Marquet et al. (2011) recovered more than 97% of receptor-fluid radioactivity as unchanged BPA in human and rat skin [49], while Reale et al. (2021) found BPA-glucuronide below the limit of quantification after 24 h [52]. For BPS, Liu and Martin (2019) observed approximately 20% conjugation in reconstructed epidermal tissue despite limited receiver-side metabolism [50] whereas Marquet et al. (2024) reported that less than 10% of permeated BPS was recovered as glucuronide or sulfate conjugates [30]. Cutaneous metabolism therefore varied across experimental systems and skin compartments, emphasizing the need to consider tissue-associated metabolism alongside receptor-fluid measurements when assessing bisphenol disposition.

Species differences in skin permeation complicate the quantitative extrapolation of animal data to humans. Marquet et al. (2011) reported ex vivo maximum BPA fluxes of 1.48 µg/cm²/h in rat skin and 0.12 µg/cm²/h in human skin, representing an approximately 12-fold difference under matched conditions [49]. A subsequent comparison found approximately threefold higher BPA flux in rat than human skin when BPA was applied in acetone [48]. Zalko et al. (2011) also observed greater BPA metabolism in viable pig than human skin, despite no major difference in overall absorption between models [20]. Nevertheless, animal models remain useful from a mechanistic perspective. Using a Triple-Pack approach integrating rat in vitro and in vivo data with human in vitro measurements, Marquet et al. (2025) estimated human in vivo BPS dermal absorption at 1.4% of the applied dose [36]. This represents an extrapolated estimate rather than directly measured human absorption and should not be generalized across exposure conditions.

**Fig. 2:**
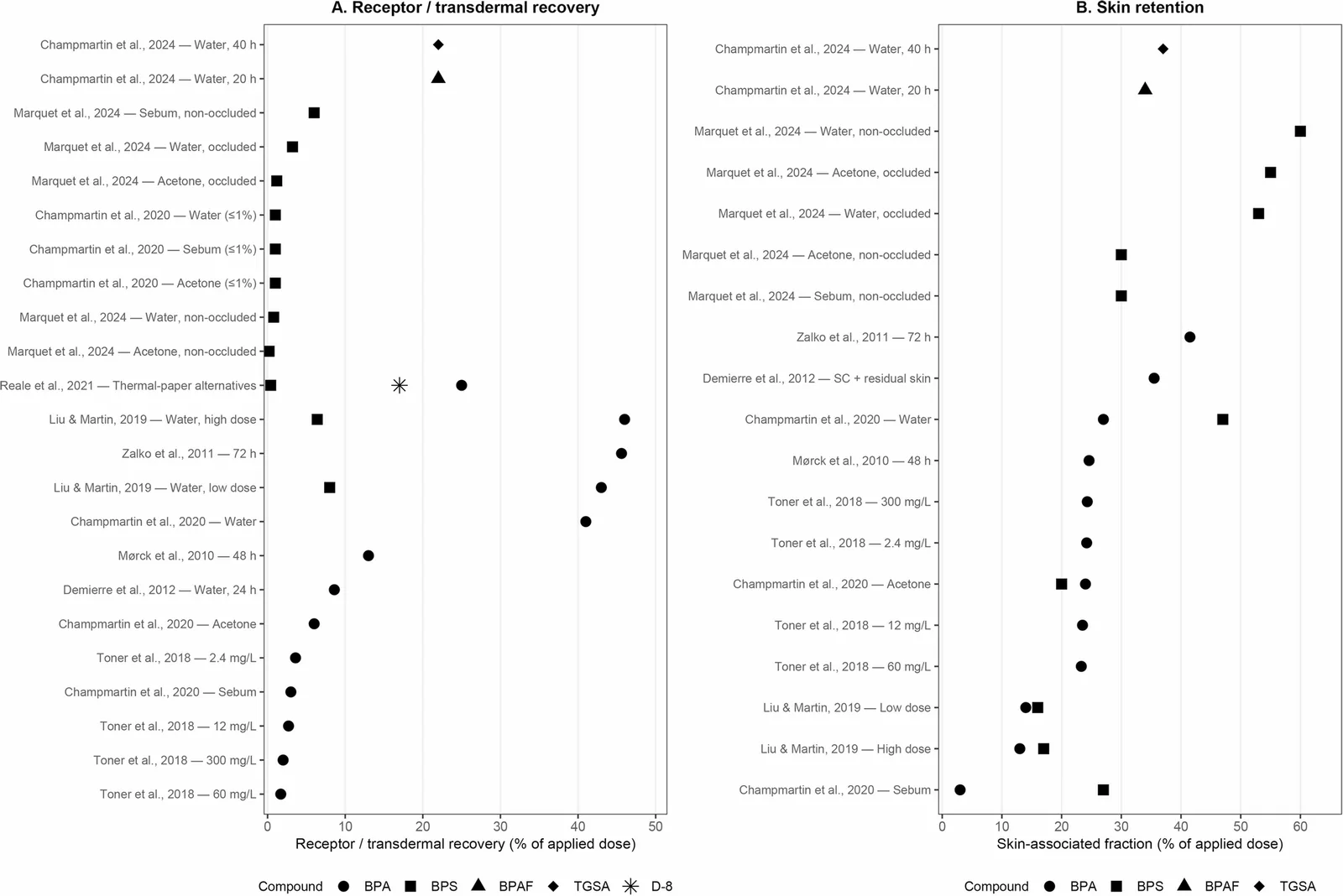
Comparative dermal disposition of bisphenols and analogs across human in vitro and ex vivo skin models. (**A**) Receptor-fluid or transdermal recovery expressed as a percentage of the applied dose. (**B**) Skin-associated fraction expressed as a percentage of the applied dose, based on reported or derived compartment-specific measurements. Each point represents an experimental condition rather than an independent study, with multiple conditions retained to illustrate variability across vehicles, doses, and skin models. Values derived from Marquet et al. (2024) include calculated receptor-recovery percentages and approximate skin-retention estimates based on reported distributions. PF201 from Reale et al. (2021) was excluded because receptor concentrations were below the limit of quantification

**Fig. 3:**
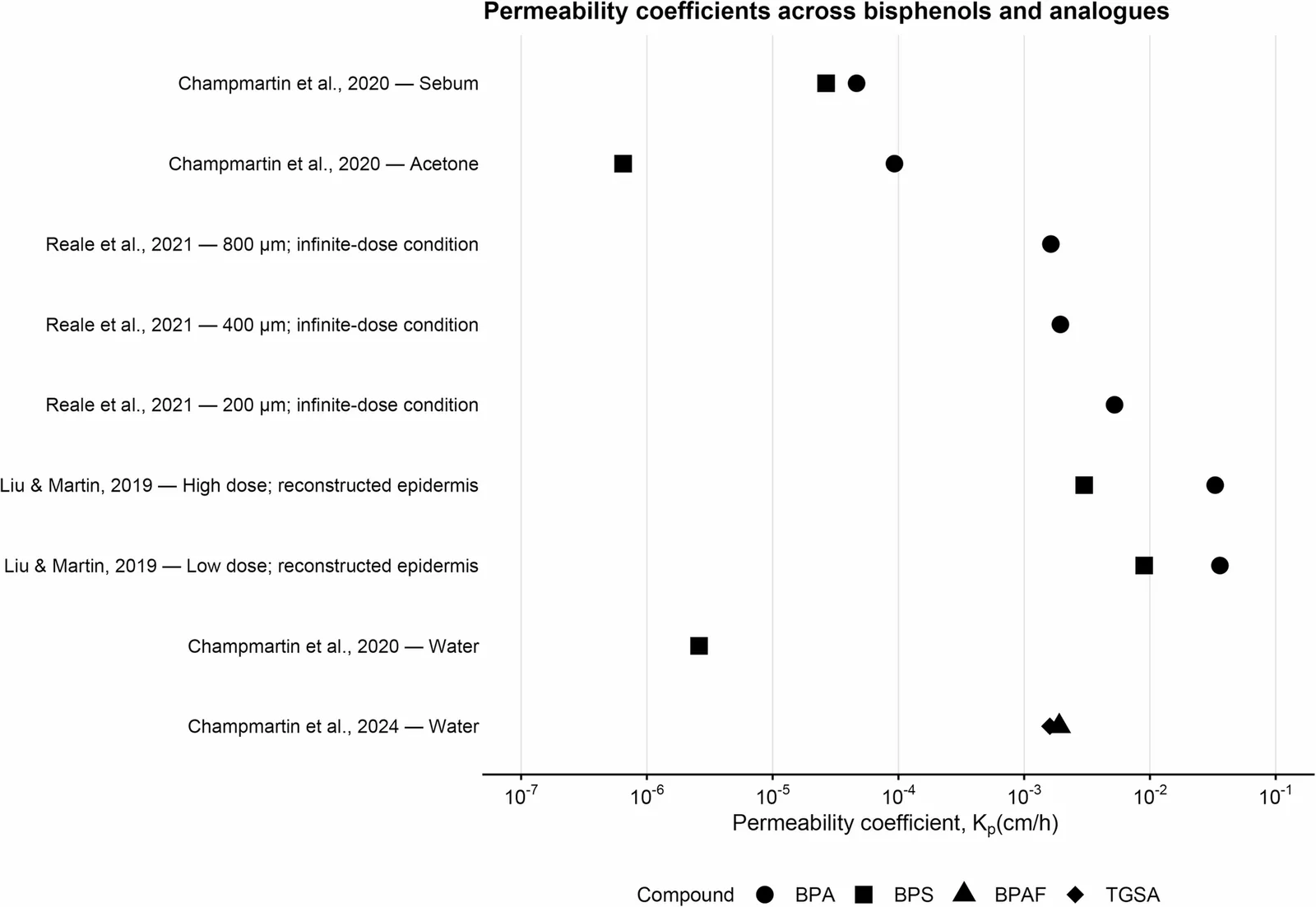
Reported permeability coefficients (Kp) for BPA and selected analogs across human dermal models. Values are displayed on a logarithmic scale. Individual points represent experimental conditions rather than independent studies. Differences in skin model, thickness, vehicle, and dosing conditions should be considered when comparing absolute values

**Fig. 4:**
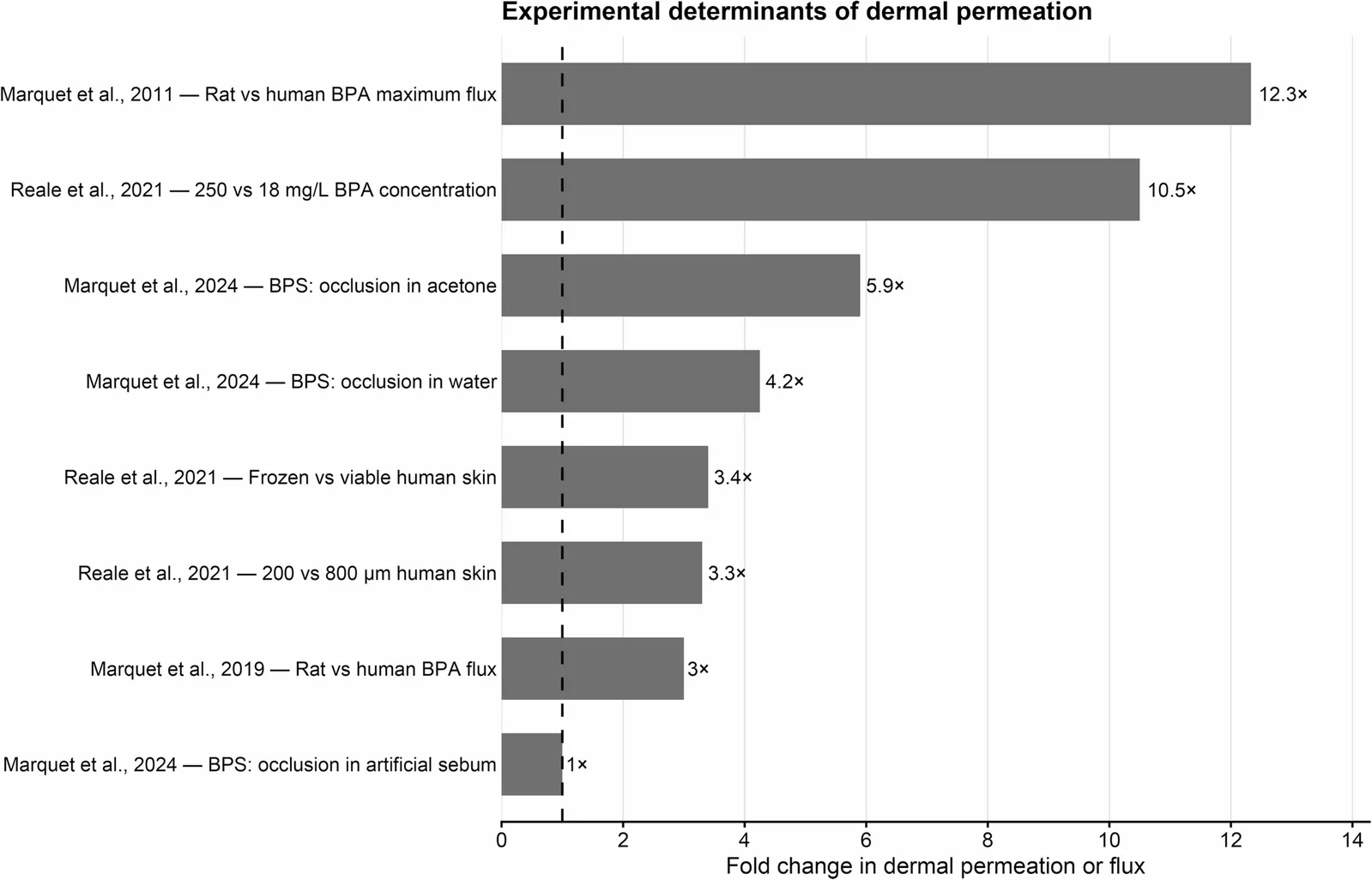
Experimental determinants of bisphenol dermal permeation. Fold changes represent within-study comparisons of permeability or flux associated with skin preparation, membrane thickness, concentration, species, or occlusion. Values are descriptive effect ratios and were not statistically pooled across studies

Recent evidence indicates that BPA substitutes do not share a uniform dermal permeability profile. Champmartin et al. (2024) reported approximately 22% and 23% absorption of BPAF and TGSA through fresh human skin after 20 and 40 h, respectively, with substantial skin retention [47]. Their permeability coefficients (Kp) were 1.9 × 10⁻³ and 1.6 × 10⁻³ cm/h, respectively, indicating appreciable permeation compared with the relatively low permeability reported for BPS under tested conditions [47]. To place these compound-specific findings within the broader experimental evidence, a descriptive quantitative visual synthesis was conducted to compare receptor-fluid recovery and skin retention (Fig. 2), permeability coefficients (Fig. 3), and the influence of experimental conditions on dermal permeation (Fig. 4). Individual experimental conditions were presented without statistical pooling to preserve differences in study designs, exposure conditions, and measured endpoints.

The descriptive quantitative synthesis illustrates substantial variability in bisphenol dermal disposition across compounds and experimental conditions (Figs. 2, 3 and 4). Receptor-fluid recovery and skin retention showed distinct patterns, with BPA generally exhibiting greater transdermal passage than BPS, while BPS demonstrated appreciable cutaneous retention (Fig. 2). Permeability coefficients varied considerably across experimental models, with BPAF and TGSA exhibiting appreciable permeability compared with BPS under the conditions evaluated (Fig. 3). Within-study comparisons further demonstrated the influence of skin preparation, thickness, applied concentration, species, and occlusion on measured permeation (Fig. 4). These differences limit direct comparison of absolute absorption estimates and support the use of compound- and condition-specific parameters rather than a uniform dermal absorption fraction.

### In Vivo Toxicokinetics and Controlled Human Dermal Exposure Studies

While in vitro and ex vivo studies characterize skin permeation and retention, in vivo investigations provide insight into systemic absorption kinetics, bioavailability, and subsequent disposition following dermal exposure. Animal experiments and controlled human studies offer complementary evidence, although differences in exposure conditions and potential contributions from multiple exposure routes complicate interpretation. Isotope-labeled human studies provide more direct evidence of route-dependent toxicokinetics, demonstrating delayed uptake and prolonged systemic exposure following dermal BPA administration, while evidence for its substitutes remains more limited and compound-dependent [18, 28, 53].

Animal in vivo studies provide evidence of systemic uptake and post-exposure disposition, although findings require careful interpretation across doses, vehicles, and species. Marquet et al. (2011) demonstrated continued BPA absorption following an 8-h dermal exposure in rats, with skin-associated residues declining from approximately 33 to 6 µg/cm² while cumulative absorbed dose increased from 25 to 46 µg/cm² over the subsequent 64 h, supporting the role of skin as a temporary depot [49]. In Swiss albino mice, Singh et al. (2023) reported peak serum unconjugated BPA concentrations of approximately 745 ng/mL at 24 h following a single high topical dose (250 mg/kg body weight in acetone) [54]. However, these high-dose findings cannot be directly extrapolated to typical human exposure conditions. Other animal investigations primarily examined toxicodynamic outcomes: Malaisé et al. (2021) reported altered offspring immune responses following repeated maternal dermal exposure to BPA, BPS, and BPF without measuring systemic bioavailability [55], whereas Lee et al. (2022) documented BPF skin penetration and sensitization rather than systemic toxicokinetic profiles [56].

Controlled receipt-handling studies demonstrate increased internal BPA exposure under defined contact conditions, although attribution to the dermal route varies with study design. Ehrlich et al. (2014) reported an increase in specific gravity-adjusted urinary BPA from a geometric mean of 1.8 µg/L at baseline to 5.8 µg/L at 4 h following two hours of receipt handling without gloves. In a subset of 12 participants providing sequential samples, concentrations reached 11.1 µg/L at 8 h and remained elevated at 24 h, whereas no statistically significant increase was observed during the nitrile-glove condition [57]. Hormann et al. (2014) examined receipt handling after hand sanitizer use followed by consumption of food touched with contaminated hands. Among six participants analyzed using blood collected from the vein draining the exposed hand, mean peak unconjugated serum BPA reached 7.19 ± 1.26 ng/mL, while total urinary BPA reached 25.77 ± 8.56 µg/g creatinine at 90 min [16]. Because this protocol combined dermal contact with potential oral and oral-mucosal uptake, and serum measurements reflected local venous drainage, the observed concentrations cannot be attributed exclusively to dermal absorption or interpreted as representative systemic concentrations.

Isotope-labeled human studies provide stronger evidence of route-dependent BPA toxicokinetics by distinguishing administered BPA from background exposure. Liu and Martin (2017) reported urinary BPA-d16 peaks 15–34 h after simulated receipt handling among five participants with detectable excretion, compared with 1–5.5 h following dietary administration. Labeled BPA remained detectable in urine one week later in three participants and for up to nine days in a single extended follow-up [18]. Unconjugated BPA-d16 was also detected in serum at 22 and 51 h in that participant, although these observations cannot be directly compared with the oral arm because the serum sampling windows differed. In a separate study, Sasso et al. (2020) administered BPA-d6 dermally to ten volunteers at 100 µg/kg body weight over 12 h and estimated systemic bioavailability at approximately 2.2%. Unconjugated BPA accounted for 8.81% of total serum BPA area under the curve (AUC), compared with 0.56% in a previously published oral study [28]. The apparent terminal half-lives of 17.6 h for unconjugated BPA and 21.4 h for total BPA were influenced by continued absorption from the dermal compartment rather than necessarily reflecting slower systemic clearance.

Human toxicokinetic evidence for BPS remains limited and indicates differences from BPA. Liu and Martin (2019) investigated five participants handling BPS-containing thermal receipts and reported maximum urinary BPS concentrations of 0.93–3.0 ng/mL, although serum BPS remained below detection during the initial 7.5-h monitoring period [50]. The reported unconjugated fraction of 6.9 ± 2.8% represented the proportion of free BPS relative to total BPS in postexposure urine samples with detectable unconjugated BPS, rather than an average across all participants or collected samples. In a controlled dermal dosing study, Khmiri et al. (2020) applied 1 mg/kg body weight of deuterated BPS to 40 cm² of forearm skin in six volunteers for 6 h. Plasma concentrations were below the lower limit of quantification at most sampling times, while urinary recovery over 72 h averaged 0.004 ± 0.003% of the applied dose as unchanged BPS-d8 and 0.09 ± 0.07% as BPS-glucuronide [53]. These low, analyte-specific urinary recoveries do not represent complete dermal bioavailability and should not be used to infer the prolonged systemic kinetics observed for BPA.

These route-dependent kinetics have important implications for occupational biomonitoring. Following dermal BPA exposure, urinary excretion may continue well beyond the contact period, such that immediate post-shift sampling may precede peak excretion, while pre-shift measurements may reflect residual uptake from earlier exposures [18, 28]. Interpretation of urinary bisphenol concentrations therefore requires consideration of exposure timing, contact and washing history, potential dietary or hand-to-mouth contributions, and whether the measured analyte represents unconjugated or total bisphenol. These considerations are particularly relevant when evaluating real-world exposure associated with repeated thermal paper handling.

### Real-World Exposure: Thermal Paper and Occupational Handling

Thermal paper handling represents one of the best-characterized real-world scenarios of potential dermal exposure to BPA and its substitutes, as thermal paper developers are commonly present as non-polymer-bound, surface-accessible compounds that can transfer to skin during contact [8, 17, 26]. Evidence from product surveys, experimental transfer studies, controlled handling investigations, and occupational biomonitoring provides complementary information on chemical occurrence, skin loading, and internal exposure. However, detection in paper and modeled intake do not directly establish systemic absorption, while occupational biomonitoring alone cannot isolate the dermal contribution.

Product-occurrence surveys identify thermal receipts as an important source of BPA and, in some markets, BPS among the paper products examined. Liao and Kannan (2011) detected BPA in 94% of 103 thermal receipts and estimated that receipts accounted for more than 98% of modeled BPA intake from the paper products included in their assessment [26]. Subsequent surveys from Belgium, China, Nigeria, Brazil, and the United Arab Emirates also documented BPA in thermal receipts, with BPS additionally identified in Brazilian samples [9, 58–62]. Studies incorporating receipt-handling scenarios generally estimated higher dermal intake among frequent occupational handlers than in general-population scenarios, although these estimates depend on measured paper concentrations and assumptions regarding contact frequency, transfer efficiency, and dermal absorption rather than direct measurements of systemic uptake [26, 58–61].

Experimental studies demonstrate that BPA can transfer rapidly from thermal paper to skin, with the amount deposited influenced by contact conditions. Biedermann et al. (2010) measured approximately 1 µg of BPA transferred to the forefinger and middle finger combined within 5 s of receipt handling, with roughly tenfold greater transfer to wet or greasy skin [17]. Transferred BPA remained largely extractable from the skin after 2 h, although these measurements did not establish transdermal absorption. Fan et al. (2015) further demonstrated pressure-dependent BPA transfer from thermal paper to other paper materials, approximately twofold faster migration into synthetic sweat than water, and residual BPA on hands (19–47%) following the washing procedures evaluated [63]. In contrast, Russo et al. (2017), Yang et al. (2019), and Shin et al. (2026) measured bisphenol concentrations in paper products and estimated dermal exposure using assumed transfer and absorption parameters rather than directly measuring paper-to-skin transfer [44, 64, 65]. Notably, Shin et al. (2026) analyzed Korean paper samples collected in 2015, so their findings characterize an earlier period of BPA substitution rather than contemporary market conditions [44].

Occupational biomonitoring studies associate thermal receipt handling with increased internal bisphenol exposure, although pre- and post-shift changes vary across studies and compounds. Thayer et al. (2016) reported a significant increase in total urinary BPS among cashiers handling BPS-containing receipts (0.23 to 0.54 µg/g creatinine; *p* < 0.001), whereas the increase in urinary BPA among those handling BPA receipts was not statistically significant (*p* = 0.10), despite higher post-shift concentrations than in non-cashiers [22]. Stronger evidence for a contribution from hand contact was provided by Lee et al. (2018), whose two-week crossover study of 54 female cashiers found approximately twofold higher post-shift urinary BPA without gloves, but no significant change during glove use [66]. Jang et al. (2022) independently demonstrated rapid BPA and BPS transfer to fingertips during receipt handling, although these surface measurements did not establish systemic absorption [67]. In French cashier studies, Ndaw et al. (2016) observed significantly higher total urinary BPA in cashiers than controls without a corresponding significant difference in unconjugated BPA [22], while Ndaw et al. (2018) reported higher total urinary BPS in cashiers, including significant pre- to post-shift increases [24]. In a simulated handling study, Lv et al. (2017) observed approximately threefold higher urinary BPA and an association with urinary 8-hydroxy-2′-deoxyguanosine (8-OHdG), an oxidative DNA damage marker [25]. However, this association does not establish causality, and the study’s estimated dermal contribution should not be interpreted as a directly measured route-specific absorption fraction.

Occupational exposure in BPA manufacturing and related industries presents a more complex exposure profile than receipt handling, with the relative importance of dermal contact and inhalation varying across workplace conditions. Heinälä et al. (2017) reported elevated urinary BPA among thermal-paper coating workers in Finland despite generally low airborne concentrations, suggesting an important contribution from skin contact in these tasks [68]. In contrast, three studies by Hines et al. examined complementary urinary, air, and hand-wipe measurements from the same US manufacturing worker cohort rather than independent study populations [69–71]. Urinary total BPA concentrations were approximately 70-fold higher than those in the contemporaneous US adult population [69]. In the integrated analysis, end-shift urinary BPA correlated strongly with both airborne BPA (*r* = 0.79) and hand-wipe concentrations (*r* = 0.75), with air remaining the stronger predictor when both measures were included in the regression model [71]. These contrasting findings suggest that exposure pathways differ across industrial processes, although associations between external contamination and urinary BPA cannot quantify the absorbed contribution of either route.

Occupational biomonitoring findings were not uniformly positive across exposure settings. In simulated receipt-handling experiments involving three volunteers, Porras et al. (2014) reported urinary BPA concentrations that generally remained within non-occupational background ranges [72]. Porras et al. (2024) similarly found urinary BPA and BPF concentrations comparable to background levels among sewage-pipe relining and floor-coating workers, although elevated BADGE and BFDGE metabolites in some workers indicated exposure to these epoxy-related compounds [73]. These differences highlight the importance of exposure matrix, analyte selection, contact conditions, and biomonitoring timing when interpreting occupational findings [17, 63]. Absence of an elevated parent-bisphenol biomarker does not necessarily exclude dermal uptake of related compounds.

### Dermal Contact from Textiles, Wearables, and Consumer Materials Studies

Beyond thermal paper, textiles, wearables, hygiene products, diapers, school supplies, and contaminated dust represent additional potential sources of dermal bisphenol exposure. These materials involve distinct contact conditions, including prolonged wear, repeated handling, moisture, and occlusion. However, much of the available evidence concerns chemical occurrence, migration, or modeled exposure rather than directly measured systemic uptake.

Textile studies have documented BPA and its analogs in infant clothing, children’s socks, pantyhose, and maternity garments, with considerable variation in concentrations and modeled dermal exposure [37, 38, 42]. Xue et al. (2017) detected BPA in 82% of 77 textile samples, with a mean concentration of 366 ng/g and a maximum of 13,300 ng/g in polyester infant socks; estimated dermal doses reached 7,280 pg/kg body weight/day [37]. Freire et al. (2019) reported BPA concentrations up to 3,736 ng/g in children’s socks, with a modeled median exposure of 17.6 pg/kg body weight/day among children aged 36–48 months [38]. Herrero et al. (2023) found BPA in all 120 garments examined, with a median concentration of 7.43 ng/g and higher concentrations in polyester-containing clothing [42]. Notably, Li and Kannan (2018) reported a median BPS concentration of 1,430 ng/g in pantyhose, reaching 2.2 mg/g in certain high-spandex samples, with estimated dermal exposure up to 45,900 pg/kg body weight/day [74]. However, differences in fabric composition and extraction procedures complicate comparisons; complete fabric dissolution yielded concentrations up to 286-fold higher than ultrasonic extraction for certain analytes [74]. Beyond BPA, textile surveys also identified BPS, BPF, and BPB, although their occurrence varied across products and studies [37, 41–43]. These studies estimated dermal exposure by combining measured textile concentrations with modeled migration, penetration, and contact parameters rather than directly measuring systemic uptake. Their exposure estimates are therefore relevant to dermal assessment but remain conditional on the assumptions examined in the subsequent modeling section.

Beyond chemical occurrence, laundering and moisture influence bisphenol availability for dermal exposure. Wang et al. (2019) reported higher median BPA concentrations in used than new clothing (34.2 versus 17.7 ng/g) and demonstrated redistribution between garments during simulated laundering [39]. In artificial-sweat experiments involving three selected used garments, BPA migration ranged from 0.049 to 0.308 ng/cm²/day. Assuming 46% dermal penetration, estimated exposure ranged from 6.07 to 38.3 ng/kg body weight/day for adults and 9.34 to 58.8 ng/kg body weight/day for toddlers, compared with modeled dry-contact estimates of 0.006–0.089 and 0.009–0.137 ng/kg body weight/day, respectively [39]. Jurikova et al. (2024) similarly measured migration into simulated sweat from four selected textiles and estimated BPA exposures of 0.223–114 ng/kg body weight/day under assumed penetration fractions of 10% or 46%; the highest modeled dry-textile estimate was 0.209 ng/kg body weight/day [43]. Although these findings indicate substantially greater potential exposure under the wet-contact scenarios, the differences reflect both experimentally determined migration and distinct assumed penetration parameters. The resulting estimates are not measured systemic doses, and comparisons with oral health-based benchmarks require route-specific interpretation.

Beyond textiles, school supplies, hygiene products, and diapers represent additional potential dermal exposure sources with distinct contact conditions. Ding et al. (2022) detected 15 bisphenols in school supplies, with the highest median summed concentration in paper products (1,347 ng/g) and modeled median dermal intakes of 157 and 433 ng/day for general and occupational scenarios, respectively [41]. Intimate-contact products introduce different exposure considerations. Gao and Kannan (2020) detected bisphenols in feminine hygiene products and estimated median dermal absorption doses of 0.039, 0.097, and 0.064 ng/kg body weight/day for pads, panty liners, and tampons, respectively, using an assumed 5% absorption fraction [40]. These estimates warrant caution because pads and liners primarily involve external skin contact, whereas tampons involve vaginal mucosal exposure; the study applied an assumed absorption fraction rather than directly measuring tissue- or compound-specific uptake [40]. Chabowska et al. (2023) detected BPA and related compounds in disposable diapers, reporting 95th-percentile modeled dermal doses reaching 172 ng/kg body weight/day for BPA and 412 ng/kg body weight/day for the BPA derivative BADGE·2HCl under specific diaper-size and body-weight assumptions [45]. Indoor dust provides a further potential contact source, although existing studies primarily characterize contamination and modeled exposure rather than direct dermal uptake [10, 75]. Across these matrices, estimated internal doses remain dependent on product-specific migration, contact conditions, and assumed penetration, rather than directly measured systemic absorption.

### Dermal Exposure Modeling and Risk Assessment

Quantitative models translate bisphenol concentrations in consumer materials and external contact conditions into estimates of dermal intake, systemic exposure, and potential risk. The evaluated studies encompass deterministic exposure equations, probabilistic assessments, and PBPK models. Contact-based models estimate intake from product concentrations and exposure parameters, whereas PBPK models characterize disposition following route-specific input. Although these approaches are complementary, their predictions depend on the empirical basis of chemical transfer, skin absorption, and systemic kinetic parameters. Estimated external exposure, absorbed dose, and circulating unconjugated bisphenol therefore represent distinct endpoints.

Early thermal-paper assessments relied on deterministic equations combining chemical concentrations with assumed transfer coefficients, handling frequency, contact duration, and dermal absorption fractions [9, 26, 58–62, 76]. A commonly used formulation, adapted from Liao and Kannan (2011), is:

1 $$\:EDI\:=\:k\:x\:C\:x\:HF\:x\:HT\:x\:AF/10^{\wedge6}$$

where EDI is estimated daily intake, k is the paper-to-skin transfer coefficient, C is the bisphenol concentration in paper, HF is handling frequency, HT is handling duration, and AF is the assumed dermal absorption fraction. The conversion factor applies to the specified input and output units [26, 61]. Liao and Kannan (2011) estimated median BPA intakes of 17.5 ng/day for the general population and approximately 1,300 ng/day for occupational handlers [26], while corresponding BPS estimates were 4.18 and 312 ng/kg body weight/day [76]. Lu et al. (2013) reported substantially higher mean BPA estimates of 0.69 and 40.4 µg/day for consumers and cashiers, respectively, identifying handling duration and frequency as major sources of modeled variability [59]. The differences illustrate how measured paper concentrations and assumed contact conditions jointly determine estimated intake, rather than establishing comparable systemic doses across populations. This framework, often relying on transfer coefficients derived from Biedermann et al. (2010), was subsequently applied or adapted in many studies to estimate daily dermal intake (EDI) [13, 58–60, 62, 76, 77].

Comparable deterministic frameworks have been used to assess prolonged dermal contact with bisphenol-containing textiles [37, 38, 42]. Unlike receipt-handling models, textile assessments incorporate material surface density, exposed skin area, migration, and contact duration. A commonly used formulation is:

2 $$\:\\\begin{array}{c}EXPderm=\\\frac{C\times\:D\times\:SA\times\:F_{mig}\times\:F_{contact}\times\:F_{pen}\times\:T\times\:N}{BW}\end{array}$$

where C is the bisphenol concentration in fabric, D is textile surface density, SA is skin contact area, Fmig is the migration rate, Fcontact is the contact fraction, Fpen is the dermal penetration fraction, T is contact duration, N is exposure frequency, and BW is body weight. For dry textiles, a migration rate of 0.005 day⁻¹ and penetration fraction of 0.01 have been used as fixed assumptions [37, 43]. In contrast, Jurikova et al. (2024) assessed sweat-wet textiles using experimentally measured migration into simulated sweat and alternative penetration fractions of 0.10 and 0.46 [43]. These differences illustrate how modeled dermal exposure can change substantially with migration conditions and assumed penetration, even when the underlying textile concentrations remain unchanged. Related product-based assessments, including sanitary materials, exhibit similar sensitivity to migration and absorption assumptions [45, 78].

Behavioral observations reveal additional limitations of standardized contact assumptions. Bernier and Vandenberg (2017) observed 698 receipt-handling interactions in a college cafeteria, reporting a mean contact duration of 11.5 min; only 11% conformed to the contact-duration and skin-area assumptions of the regulatory scenario examined [79]. Their exposure estimates nevertheless relied on literature-derived bisphenol concentrations, transfer coefficients, and absorption fractions. Thus, observed contact behavior improves exposure characterization but does not independently establish the amount transferred to skin or absorbed. Similarly, experimentally observed increases in transfer following sanitizer use cannot be applied as universal multipliers for systemic absorption [16, 79].

PBPK models extend contact-based estimates by predicting internal concentrations and metabolic disposition. Mielke et al. (2011) incorporated assumed dermal BPA input into a human pharmacokinetic model, demonstrating the dependence of predicted internal concentrations on exposure and absorption assumptions [21]. Karrer et al. (2018) subsequently modeled BPA, BPS, BPF, and BPAF using compound-specific metabolic parameters, while retaining common dermal absorption fractions of 20% for thermal paper and 60% for personal care products [29]. These models also require two mechanisms to be distinguished: skin diffusion and retention can delay systemic input, whereas partial avoidance of presystemic intestinal and hepatic metabolism may alter the proportion of circulating unconjugated bisphenol [21, 28, 29]. Reduced first-pass metabolism does not itself explain delayed absorption.

Mechanistic dermal PBPK models improve exposure assessment by explicitly representing chemical transfer through skin compartments and subsequent systemic disposition. Wiśniowska et al. (2023) integrated multilayer skin and systemic PBPK models for BPA exposure from thermal paper, predicting a stratum corneum reservoir that could sustain absorption after contact [80]. However, because the external dose was reconstructed from urinary excretion, model agreement with serum measurements did not independently validate the original exposure estimate. Hu et al. (2023) combined parallel-layered skin models with small controlled human contact experiments involving BPS-containing thermal paper (*n* = 4) and personal care products containing BPF (*n* = 4) or BPAF (*n* = 5) [81]. For BPS, approximately 28% of the paper-associated amount transferred to fingers, with an estimated 96% absorption of the transferred fraction, yielding a product-based estimate of approximately 27%. Corresponding bioaccessibility estimates were approximately 32% for BPF and 12% for BPAF under their respective experimental conditions. These studies illustrate the contribution of mechanistic modeling while demonstrating the importance of distinguishing measured transfer, estimated absorption, and reconstructed exposure inputs.

Population-level probabilistic models have produced different estimates of dermal contributions under their respective assumptions. Fu et al. (2016) estimated a relatively limited dermal contribution to selected population-level BPA outcomes [82]. In contrast, Karrer et al. (2019) combined probabilistic exposure assessment with compound-specific PBPK models for BPA, BPS, BPF, and BPAF, estimating mean potency-weighted cumulative internal exposures of 3.8 and 2.1 ng/kg body weight/day for the periods before and after the restrictions considered [83]. The uncertainty intervals overlapped, and the upper uncertainty bound increased after substitution, reflecting less certain exposure information for replacement compounds. These results point to the influence of exposure pathways, model inputs, and compound-specific assumptions on estimated internal burdens rather than establishing a universal dermal contribution.

Probabilistic and product-based models also demonstrate the sensitivity of risk characterization to exposure assumptions and toxicological benchmarks. Akcetin et al. (2025) estimated mean occupational dermal intakes of 0.0223 µg/kg body weight/day for BPA and 0.00195 µg/kg body weight/day for BPS from thermal receipts in Türkiye, alongside probabilistic exposure and hazard estimates [77]. Assessments of textiles, diapers, and sanitary products likewise showed sensitivity to migration and penetration assumptions [43, 45, 78]. However, lowering a health-based benchmark changes the resulting hazard quotient without changing the modeled exposure or relative contribution of exposure routes. Comparisons with the EFSA (2023) BPA tolerable daily intake of 0.2 ng/kg body weight/day require consistent units, alignment of external, absorbed, and oral-equivalent doses, and appropriate route-specific toxicokinetic considerations [84]. Hazard indices additionally require justification for combining chemicals and endpoints; the BPA benchmark cannot automatically be applied to its substitutes.

In vitro–in vivo extrapolation provides complementary evidence for compound-specific dermal absorption. Knudsen et al. (2015) estimated up to approximately 6% human dermal bioavailability for tetrabromobisphenol A (TBBPA), a brominated BPA analog, using comparative human and rat experiments and the parallelogram method [85]. As with PBPK predictions, such estimates depend on the underlying experimental evidence and extrapolation assumptions. More broadly, modeled internal exposure depends on successive processes involving material-to-skin transfer, contact frequency and area, skin penetration and retention, and systemic disposition, rather than external contact alone. The relative influence of these parameters varies with compound, exposure scenario, and model structure [77, 81]. Reliable parameterization therefore requires condition-specific information on skin preparation, donor number, dose, vehicle, occlusion, wash-off, exposure duration, receptor conditions, and compartment-specific recovery, alongside permeability, flux, and lag-time measurements. Distinguishing measured, calculated, fitted, and assumed inputs is essential when extrapolating across bisphenols and exposure scenarios.

### Substitution and Regulatory Interpretation

The combined evidence from chemical occurrence, dermal absorption studies, occupational biomonitoring, and exposure modeling raises important questions about bisphenol substitution and regulatory risk assessment. The replacement of BPA in thermal paper has altered the mixture of chemicals available for dermal exposure without necessarily reducing overall exposure potential or toxicological concern. Lee et al. (2021) documented BPA and BPS in Korean receipts, with modeled occupational intakes reaching 45,123 and 52,065 ng/day, respectively [86]. Yang et al. (2019) identified additional thermal-paper developers, including D-90, D-8, BPS-MAE, and TGSA [65], while subsequent surveys continued to detect BPS and other alternatives [44, 61, 77]. Han et al. (2024) further detected chlorinated BPS derivatives in thermal and other paper products [87]. Bisphenol occurrence in currency and recycled-paper products suggests that exposure sources may extend beyond receipts, although the available findings do not establish that recycling itself generates these derivatives [65, 87, 88]. Nasello et al. (2022) additionally demonstrated BPA migration from discarded thermal paper into soil and water, alongside modeled human dermal exposure estimates [89]. Importantly, substitution changes both exposure composition and toxicokinetic considerations: BPS generally exhibits lower transdermal passage than BPA but substantial skin retention, whereas BPAF and TGSA demonstrate appreciable penetration under tested conditions [27, 47, 50]. Neither the presence of alternatives nor a BPA-free designation therefore establishes reduced dermal exposure or overall toxicological risk.

Regulatory interpretation requires distinguishing exposure estimates, toxicological benchmarks, and the rationale for restrictions. EFSA’s BPA tolerable daily intake (TDI) changed from the historical value of 50 µg/kg body weight/day to a temporary TDI of 4 µg/kg body weight/day in 2015 and 0.2 ng/kg body weight/day in 2023 [84]. During the EU thermal-paper restriction process, ECHA’s Committee for Risk Assessment (RAC) concluded that worker risks from BPA-containing thermal paper were inadequately controlled, whereas the Committee for Socio-economic Analysis (SEAC) questioned whether the socioeconomic benefits justified the costs [15, 90, 91]. The EU subsequently restricted the placing on the market of thermal paper containing BPA at concentrations of 0.02% by weight or greater after 2 January 2020 [15]. Controlled human and glove-intervention studies provide additional evidence of internal BPA uptake and route-dependent kinetics [18, 28, 66]. Although earlier assessments generally reported relatively low dermal exposure under their assumptions and applicable benchmarks [13], more recent modeling illustrates how updated guidance can alter screening interpretations [61, 77]. Such comparisons require alignment of external, absorbed, and oral-equivalent doses; lowering a TDI changes the estimated hazard quotient, not the underlying exposure. Furthermore, BPA-derived benchmarks cannot be directly applied to replacement bisphenols without compound-specific toxicological justification.

### Limitations of the Current Evidence Base

Human dermal toxicokinetic evidence remains concentrated on BPA and a limited number of BPS studies, typically involving small samples and single exposure episodes [18, 28, 50, 53]. Consequently, compound-specific systemic kinetics under repeated, low-dose exposure remain insufficiently characterized, particularly for other bisphenol analogs. The fate of chemicals retained in skin also requires clarification, including whether residues contribute to continued absorption after washing or remain skin-associated without further systemic uptake. Occupational biomonitoring provides evidence of exposure but rarely distinguishes dermal absorption from dietary, inhalation, or hand-to-mouth contributions [24, 92]. Future studies should integrate realistic material-to-skin transfer, repeated contact, wash-off experiments, and time-resolved, route-discriminating biomonitoring of parent compounds and metabolites.

Comparisons across experimental studies are further constrained by differences in donor numbers, skin preparation and integrity, tissue thickness and viability, dose, vehicle, occlusion, receptor conditions, and outcome definitions [20, 51, 52]. High-dose or solvent-based experiments provide mechanistic information but may not reproduce typical consumer contact. For textiles and other intimate-contact materials, estimated doses frequently depend on assumed migration and penetration parameters rather than directly measured systemic uptake [42, 43, 45, 78, 81]. Priority research should therefore examine repeated exposure, sweating, occlusion, altered skin barriers, and mixtures under realistic conditions, while improving route-specific evidence linking exposure to health outcomes [25, 92]. Consistent reporting of compartment-specific mass balance, permeability coefficients, flux, lag time, and interindividual variability would also improve cross-study interpretation and PBPK parameterization.

### Limitations of this Review

This review was limited to English-language peer-reviewed studies and was not prospectively registered. Screening was performed by one reviewer with a second-reviewer cross-check rather than independent duplicate screening. Although study-specific weaknesses were considered, no formal risk-of-bias, certainty, or publication-bias assessment was conducted. Heterogeneous outcomes and incomplete variance reporting precluded meta-analysis, limiting quantitative generalization across compounds and conditions.

## Conclusions

Converging experimental and controlled human evidence establishes dermal uptake of BPA, with occupational studies supporting its relevance under frequent-contact conditions. The magnitude and timing of uptake depend on material transfer, skin and vehicle conditions, and retention, making receptor-fluid recovery alone an incomplete measure of dermal disposition. BPS generally exhibits lower transdermal passage than BPA under comparable experimental conditions, but neither lower permeability nor chemical substitution establishes reduced overall risk. Thermal paper remains the best-characterized source, whereas systemic contributions from other consumer materials are less certain and rely more heavily on modeled estimates. Accurate exposure assessment requires distinguishing skin loading, retention, transdermal passage, and systemic bioavailability using compound- and condition-specific parameters supported by realistic transfer measurements.

## Key References

- Champmartin C, Marquet F, Chedik L, Décret M-J, Aubertin M, Ferrari E, et al. Human in vitro percutaneous absorption of bisphenol S and bisphenol A: a comparative study. Chemosphere. 2020;252:126525. https://doi.org/10.1016/j.chemosphere.2020.126525
  - ◦ Demonstrates lower receptor-side passage but substantial skin retention of BPS relative to BPA in excised human skin, highlighting the importance of vehicle conditions and compartment-specific recovery in dermal absorption assessment.
- Sasso AF, Pirow R, Andra SS, Church R, Nachman RM, Linke S, et al. Pharmacokinetics of bisphenol A in humans following dermal administration. Environ Int. 2020;144:106031. https://doi.org/10.1016/j.envint.2020.106031
  - ◦ Provides controlled human toxicokinetic evidence using isotope-labeled BPA, characterizing systemic bioavailability, delayed dermal input, and the circulating unconjugated fraction under a defined exposure protocol.
- Marquet F, Champmartin C, Seiwert C, Aubertin M, Viton S, Chedik L, et al. Human in vitro percutaneous absorption of bisphenol S: assessment of the skin reservoir and occlusion effects. Toxicol In Vitro. 2024;99:105886. https://doi.org/10.1016/j.tiv.2024.105886
  - ◦ Demonstrates substantial BPS retention in human skin after surface washing and increased absorption under occlusion, suggesting a potential cutaneous reservoir that may contribute to continued exposure.
- Champmartin C, Seiwert C, Aubertin M, Joubert E, Marquet F, Chedik L, et al. Percutaneous absorption of two bisphenol A analogues, BPAF and TGSA: novel in vitro data from human skin. Chemosphere. 2024;367:143564. https://doi.org/10.1016/j.chemosphere.2024.143564
  - ◦ Provides human-skin permeation and retention data for BPAF and TGSA, demonstrating compound-specific differences that challenge extrapolation of BPS absorption behavior to other BPA alternatives.
- Thayer KA, Taylor KW, Garantziotis S, Schurman SH, Kissling GE, Hunt D, et al. Bisphenol A, bisphenol S, and 4-hydroxyphenyl 4-isoprooxyphenylsulfone (BPSIP) in urine and blood of cashiers. Environ Health Perspect. 2016;124:437–444. https://doi.org/10.1289/ehp.1409427
  - ◦ Links thermal-receipt composition with occupational biomonitoring, demonstrating significant within-shift increases in urinary BPS while highlighting compound-specific differences in biomarker responses and limitations of exposure-route attribution.
- Wiśniowska B, Linke S, Polak S, Bielecka Z, Luch A, Pirow R. Physiologically based modelling of dermal absorption and kinetics of consumer-relevant chemicals: a case study with exposure to bisphenol A from thermal paper. Toxicol Appl Pharmacol. 2023;459:116357. https://doi.org/10.1016/j.taap.2022.116357
  - ◦ Integrates multilayer skin and systemic PBPK modeling, illustrating the importance of skin retention and delayed absorption while distinguishing reconstructed exposure inputs from independently measured dermal doses.
- Wang L, Zhang Y, Liu Y, Gong X, Zhang T, Sun H. Widespread occurrence of bisphenol A in daily clothes and its high exposure risk in humans. Environ Sci Technol. 2019;53(12):7095–7102. https://doi.org/10.1021/acs.est.9b02090
  - ◦ Demonstrates BPA occurrence in everyday clothing, redistribution during laundering, and migration into artificial sweat. Highlights how clothing use and moisture influence exposure potential, while distinguishing measured migration from estimated systemic uptake.

## Supplementary Information

Below is the link to the electronic supplementary material.

Supplementary Material 1

## Data Availability

No datasets were generated or analysed during the current study.
