# Supplementary material for: Dermal Exposure to Bisphenols and Their Analogs: A Systematic Review and Narrative Synthesis
Source: Curr Environ Health Rep. 2026 Nov 13;13(1):53. doi: 10.1007/s40572-026-00574-2 (PMC13768286; doi:10.1007/s40572-026-00574-2)
Supplement: Supplementary file 1 [file 40572_2026_574_MOESM1_ESM.docx]

**Supplemental Material**

**S-1: Search Strings**

Scopus String

TITLE-ABS-KEY((bisphenol* OR BPA OR BPS OR BPF OR BPAF OR BPB OR BPE OR TGSA OR "D-8" OR BPSIP OR PF201 OR "PF 201" OR Pergafast) AND (dermal* OR cutaneous* OR skin OR percutaneous* OR transdermal* OR "stratum corneum" OR "Franz cell" OR "diffusion cell" OR "artificial sweat" OR "skin reservoir"))

Web of Science String

TS=((Bisphenol* OR BPA OR BPS OR BPF OR BPAF OR "BP analogues" OR "D-8" OR "Pergafast")

AND (dermal* OR skin* OR percutaneous* OR transdermal* OR "stratum corneum" OR integument*)

AND (absorp* OR penetrat* OR permeat* OR transfer* OR "bio-accessibility" OR "skin reservoir" OR PBPK))

ScienceDirect String

Title, abstract, keywords: (bisphenol OR BPA OR BPS OR BPF OR BPAF) AND (dermal OR skin OR percutaneous OR transdermal permeation OR “skin absorption”)

Springer Link String

(bisphenol* OR BPA OR BPS OR BPF OR BPAF OR "BP analogues" OR "D-8") AND (dermal OR skin OR percutaneous OR transdermal OR "stratum corneum") AND (absorp* OR penetrat* OR permeat* OR transfer* OR "bio-accessibility" OR PBPK)

PubMed String

(bisphenol*[Title/Abstract] OR BPA[Title/Abstract] OR BPS[Title/Abstract] OR BPF[Title/Abstract] OR BPAF[Title/Abstract] OR BPB[Title/Abstract] OR BPE[Title/Abstract] OR TGSA[Title/Abstract] OR "D-8"[Title/Abstract] OR BPSIP[Title/Abstract] OR PF201[Title/Abstract] OR Pergafast[Title/Abstract]) AND (dermal[Title/Abstract] OR cutaneous[Title/Abstract] OR skin[Title/Abstract] OR percutaneous[Title/Abstract] OR transdermal[Title/Abstract] OR "stratum corneum"[Title/Abstract] OR "Franz cell"[Title/Abstract] OR "diffusion cell"[Title/Abstract] OR "artificial sweat"[Title/Abstract] OR "skin reservoir"[Title/Abstract])

Complementary contact and biomonitoring search concepts: (bisphenol* OR BPA OR BPS OR BPF OR BPAF OR BPB OR BPE OR TGSA OR "D-8" OR BPSIP OR PF201 OR Pergafast) AND (("thermal paper" OR receipt* OR cashier* OR textile* OR cloth* OR diaper* OR "personal care" OR "consumer product*") AND (biomonitor* OR urin* OR serum OR plasma OR transfer* OR migrat* OR PBPK OR pharmacokinetic* OR toxicokinetic* OR model*))

Embase String

('bisphenol a'/exp OR 'bisphenol s'/exp OR 'bisphenol f'/exp OR 'bisphenol'/exp OR bisphenol*:ti,ab) AND ('skin absorption'/exp OR 'percutaneous absorption'/exp OR 'skin exposure'/exp OR 'dermal exposure'/exp OR ((dermal* OR skin* OR percutaneous* OR cutaneous*) NEAR/3 (absorp* OR penetrat* OR permeat* OR exposur*)):ti,ab) AND ('paper'/exp OR ('thermal paper' OR receipt* OR currency OR 'paper product*'):ti,ab)

**S-2: PRISMA 2020 Checklist**

| **Section** | **Item** | **Checklist Item** | **Location in Manuscript** |
| --- | --- | --- | --- |
| Title | 1 | Identify the report as a systematic review. | Title page |
| Abstract | 2 | PRISMA 2020 for Abstracts checklist. | Abstract section |
| Introduction | 3 | Rationale for the review in the context of what is already known. | Introduction, Paragraph 1-3 |
| Introduction | 4 | Explicit statement of the objectives or questions. | End of Introduction |
| Methods | 5 | Eligibility criteria specified and justified. | Methods |
| Methods | 6 | Information sources (databases, registers, etc.) and the date of last search | Methods |
| Methods | 7 | Full search strategies for all databases. | Methods and supplemental materials S-1. |
| Methods | 8 | Process for selecting studies. | Methods |
| Methods | 9 | Data collection methods. | Methods |
| Methods | 10 | Data items and assumptions. | Methods |
| Methods | 11 | Risk of bias assessment. | No formal risk-of-bias tool applied; rationale provided in Methods. |
| Methods | 12 | Effect measures. | Methods: Descriptive study-level proportions (% applied dose), Kp, and within-study fold changes; no pooled effect measure. |
| Methods | 13 | Synthesis methods. | Methods: Narrative synthesis supplemented by descriptive quantitative visual synthesis; no meta-analysis. |
| Results | 14-21 | Flow diagram, study characteristics, results, synthesis. | Results and Discussion section, Figure 1, Supplemental Materials: S-3 to S-9 |
| Discussion | 22-23 | Interpretation of results, limitations. | Results and Discussion section |
| Other | 24 | Registration and protocol. | Not prospectively registered |
| Other | 25 | Support/funding. | Funding Section |
| Other | 26 | Competing interests. | Competing interests’ section |
| Other | 27 | Availability of data | Availability of data and materials section |

**S-3. Summary of in vitro / ex vivo studies**

| Study | Species / Model | Exposure Duration | Bisphenol(s) | % Absorbed / Receptor | % Retained in Skin | Metabolism | Key Pattern |
| --- | --- | --- | --- | --- | --- | --- | --- |
| Champmartin *et al.* (2020) | Fresh human split-thickness skin, static Franz cells | 40 h | BPA; BPS | BPA: 3% (sebum), 6% (acetone), 41% (water); BPS: ≤1% in all vehicles | BPA: 3% (sebum), 24% (acetone), 27% (water); BPS: 20% (acetone), 27% (sebum), 47% (water) | Limited; absorbed dose mostly unmetabolized | Strong vehicle effect; BPS receptor recovery much lower than BPA, but BPS accumulated markedly in skin |
| Champmartin *et al*. (2024) | Fresh human split-thickness skin, static Franz cells | TGSA: 40 h; BPAF: 20 h | TGSA; BPAF | TGSA: 22%; BPAF: 22% | TGSA: 37%; BPAF: 34% | Not assessed in this paper | Both were fast penetrants with substantial skin reservoir |
| Demierre *et al.* (2012) | Human skin, OECD TG 428 flow-through diffusion cells | 24 h | BPA | 8.6% | 0.6% residual skin membrane; 34.9% stratum corneum | Not assessed | Lower receptor recovery under realistic OECD/GLP conditions; most BPA remained on/within stratum corneum |
| Kaddar *et al*. (2008) | Pig skin, Franz static diffusion cells | 10 h (kinetics to 24 h) | BPA | 0.0% (2 h), 0.1% (5 h), 0.7% (10 h) | 3.0% (2 h), 6.9% (5 h), 11.4% (10 h) | Not assessed | Very low receptor recovery with progressive dermal accumulation |
| Liu & Martin (2019) | Reconstructed human epidermis (EpiDerm) | 25 h | BPA; BPS | BPA: 43–46%; BPS: 6.4–8.0% | BPA: 13–14%; BPS: 16–17% | Negligible/limited | BPS permeability was much lower than BPA, while both showed little skin biotransformation in this model |
| Marquet *et al.* (2011) | Ex vivo human dermatomed skin | 24 h | BPA | Human Fmax ≈ 0.12 µg/cm²/h | No accumulation during exposure | Minimal; >97% unchanged BPA in receptor fluid | Human permeability was much lower than rat; substantial inter-/intra-individual variability |
| Marquet *et al.* (2019) | In vitro rat vs human skin comparison | BPA: 48 h | BPA | NR | NR | Not assessed | Rat BPA flux was ~3-fold higher than human, so rat skin was not a reliable surrogate for human absorption of solid lipophilic BPA |
| Marquet *et al.* (2024) | Fresh human split-thickness skin, static Franz cells | 40 h | BPS | ~0.2–6% depending on vehicle/occlusion | ~30% (sebum/acetone) to ~60% (water) at 40 h | Limited; most BPS unmetabolized | Strong stratum corneum reservoir; occlusion markedly increased BPS absorption, especially in water and acetone |
| Marquet *et al.* (2025) | Rat skin, in vitro/in vivo plus IV comparison | In vitro: 40 h; in vivo: up to 72 h | BPS | In vitro: sebum 25.8%, water 2.13%, acetone 1.51%; in vivo dermal: ~10% | In vitro skin: 25–60%; in vivo skin persistence >72 h | Yes; absorbed BPS mostly unmetabolized in vitro, urinary BPS mainly glucuronidated in vivo | Marked skin retention and slow systemic release; Triple-Pack estimate for human in vivo dermal absorption: 1.4% |
| Mørck *et al.* (2010) | Full-thickness human skin, Franz diffusion cells | 48 h | BPA | 13.0% | 24.6% total skin deposition | Not assessed | Moderate percutaneous penetration with notable dermal reservoir |
| Reale *et al.* (2021) | Ex vivo human skin, flow-through diffusion cells | 24 h | BPA; BPS; D-8; PF201 | Dose Recovered (% of Applied Dose); BPA: 25%; BPS: 0.4%; D-8: 17%; PF201: <LLOQ | BPA 15 ± 14%, BPS 11 ± 6.8%, D-8 16 ± 5.4%, PF201 1.5 ± 0.9%, in skin after five tape strips (tapes not analyzed). | BPA-glucuronide <LLOQ | Skin absorption followed BPA > D-8 >> BPS > PF201; 7–12 skin samples per substance from three donors. |
| Reale *et al.* (2021) | Ex vivo human skin, flow-through diffusion cells | 24 h | BPA | 2–24% across conditions | NR | Not assessed | BPA permeation varied strongly with skin state, thickness, concentration, and vehicle volume; frozen skin and thinner skin increased flux |
| Toner *et al*. (2018) | Fresh human split-thickness skin, flow-through cells | 24 h | BPA | 1.7–3.6% | Dermal delivery 16–20%; epidermis 10–12%; dermis 3.3–6.2% | Yes, limited | Low receptor recovery but clear skin retention; some BPA-glucuronide/BPA-sulfate formed in dermis and receptor fluid, not epidermis |
| Zalko *et al.* (2011) | Viable human skin explants; viable pig ear skin; frozen pig skin control | 72 h | BPA | Human: 45.6%; pig: 65.3%; frozen pig: 58.1% | Human: 41.5%; pig: 20.8–31.9%; frozen pig: 28.8% | Yes in viable skin only | High dermal uptake; substantial skin reservoir; viable skin conjugated BPA, especially pig skin |

**S-4. Summary of In Vivo Dermal Exposure and Toxicokinetic Studies**

| Study (Year) | Species / Model | Bisphenol(s) | Exposure Design | Dose & Vehicle | Key Toxicokinetic Findings | Skin Reservoir Evidence | Additional Biological Outcomes |
| --- | --- | --- | --- | --- | --- | --- | --- |
| Lee *et al.* (2022) (published online 2021) | Dermal absorption setup + Sprague–Dawley rats for toxicity tests | BPF | Dermal absorption profiling (skin device; receptor sampling) + acute, 28-day repeat dermal, irritation, eye irritation, sensitization battery | Absorption study: 6% BPF, 100 µL applied 4 times on 0.64 cm² skin area | Absorbed through skin at 26.5%; Kp 2.2E-02 cm/h | Inferred via BPF remaining in epidermis/dermis (basis for 26.5% absorption) | No irritation/toxicological changes reported in abstract, but potent sensitization observed |
| Malaisé *et al.* (2021) | Pregnant/lactating mice; offspring assessed at PND70 | BPA, BPS, BPF | Maternal dermal exposure GD15 → PND21 (weaning) | Daily 10 µL drop on neck; 5 or 50 µg/kg bw/day in 70% ethanol | No toxicokinetic parameters reported (no plasma/urine BP kinetics); rationale notes ethanol can enhance dermal penetration | Not quantified | Sex- and dose-dependent immune alterations; e.g., changes in colonic IL-17/IFN-γ patterns in offspring |
| Marquet *et al.* (2011) | Male Sprague–Dawley rats (in vivo) + rat/human skin (ex vivo) | BPA (radiolabeled) | Occluded topical exposure in vivo (Tedlar membrane) + static Franz ex vivo | In vivo: 200 µg/cm² (from 4 mg/mL in acetone; 50 µL/cm²; acetone evaporated) | In vivo: absorption/penetration flux 2.5 ± 0.2 µg/cm²/h and rapid penetration (lag time ~0) . Ex vivo Fmax (dermatomed frozen): rat 1.48 vs human 0.12 µg/cm²/h | Skin BPA content measured after exposure: 33.0 ± 6.0 µg/cm² at end of 8 h, 6.2 ± 2.2 µg/cm² at 64 h after 8 h exposure | No irritation signal reported in the excerpted results; receptor fluid shows minimal metabolism (mostly BPA; low glucuronide) |
| Marquet *et al.* (2025) | Rats: in vitro + in vivo dermal + i.v. comparative TK | BPS ([14C]-BPS + unlabeled) | In vitro Franz on rat skin + in vivo dermal exposure (occluded; ring) + i.v. reference dosing | 20 µg/cm² applied in vitro & in vivo ; vehicles in vitro include sebum/water/acetone ; in vivo uses artificial sebum | In vitro: highest uptake in sebum (~5000 ng/cm² over 40 h) ; most absorbed unmetabolized 78–85% . In vivo dermal systemic absorption ~10% and persistence/slow release from skin (>72 h) . Triple-Pack estimate: human in vivo dermal absorption 1.4% | Significant skin reservoir: 25–60% of applied dose in vitro ; in vivo mass balance shows large fraction in exposed skin at 72 h | Urinary elimination primary (60–70%), mainly as BPS-glucuronide |
| Singh *et al.* (2023) | Female Swiss albino mice | BPA (unconjugated measured) | Single topical dose to shaved 2 cm² interscapular area | 250 mg/kg bw in acetone (200 µL) | Following 250 mg/kg topical BPA in acetone, reported serum concentrations peaked around 0.745 ng/µL at 24 h and declined thereafter; the late 168-h serum value was below the study's quantification limit. BPA remained measurable in skin at 168 h | Skin concentrations decline but persist to 168 h: 1254 ng/mg (6 h) → 8.329 ng/mg (168 h) | No dedicated toxicity endpoints beyond concentration profiling (study aim is quantification) |

**S-5: Summary of Controlled Human Dermal Exposure Studies**

| Study | Design | Sample Size | Dermal Source | Exposure Conditions | Biomarkers | Key Quantitative Findings | PK Characteristics | Notes |
| --- | --- | --- | --- | --- | --- | --- | --- | --- |
| Ehrlich *et al.* (2014) | Simulation cross-over | 24 (no gloves); 12 sequential urine; 12 gloves | Thermal receipts | 2 h handling; urine baseline & 4 h; subset urine at 8/12/24 h; repeat ≥1 week later with nitrile gloves | Urine total BPA (SG-adjusted) | GM 1.8 → 5.8 µg/L (baseline→4 h); subset peak 11.1 µg/L at 8 h; no significant increase with gloves | Delayed peak (~8 h) | Peak values are from sequential subset |
| Hormann *et al.* (2014) | Controlled experiment (multiple experiments) | Exp3: 10 (5M/5F; PK table reported for subset); Exp4: 24 (12M/12F) | Thermal receipts | Exp3: hand sanitizer + hold receipt 4 min + handle/eat fries; serial blood to 90 min; urine baseline & 90 min. Exp4: dry-hand receipt hold 4 min; blood at 30 min; urine at 60 min | Serum uBPA & conjugates; urine total BPA; hand wipes | Exp3: serum uBPA Cmax 7.19 ± 1.26 ng/mL ( higher in females), Tmax 15–90 min; BPA-G/uBPA AUC ratio 0.846 ± 0.33; urine total BPA at 90 min 25.77 ± 8.56 µg/g creatinine. Exp4: BPA on hand at 30 min ~5–6 µg; urine total BPA did not change at 60 min | Rapid early rise (Exp3) with low conjugate: free ratio; minimal change under dry-hand/short window (Exp4) | Exp3 reflects combined dermal + oral/sublingual (food contact); sanitizer acts as penetration/transfer enhancer. First-pass bypass |
| Khmiri *et al.* (2020) | Clinical TK (oral + dermal; deuterated) | 8 recruited (female); 7 oral; 6 dermal | Forearm application (not receipt handling) | Dermal: 1 mg/kg BPS-d8 applied to 40 cm² forearm for 6 h, then wiped/washed; plasma up to 48 h; urine up to 72 h | Plasma/urine BPS-d8 & BPSG-d8 | Dermal: plasma mostly <LLOQ; peak 5–8 h; urinary recovery 0.004 ± 0.003% (BPS-d8) and 0.09 ± 0.07% (BPSG-d8) of applied dose | Very low dermal absorption; delayed peak | Useful for bisphenol-analogue TK comparison |
| Liu & Martin (2017) | Controlled comparative TK: dermal vs dietary (isotope-labeled) | 6 males dermal; 5 returned for dietary; 1 repeated dermal with extended follow-up | Simulated receipt (BPA-d16) | Dermal: handle simulated receipt 5 min, then gloved 2 h, hand-wash; urine 0–48 h, serum 0–7.5 h. Follow-up (1 participant): urine 9 days, serum 22 & 51 h. Dietary (1 week later): cookie with 20 µg BPA-d16; urine 0–48 h, serum 0–7.5 h | Urine & serum free and total BPA-d16 | Dermal: urine Cmax 0.18–4.6 ng/mL, Tmax 15–34 h; 3/5 still had detectable urinary total BPA-d16 1 week later. Follow-up dermal: urine detectable 9 days, with near-linear cumulative excretion over several days | Dermal shows delayed Tmax and prolonged elimination/reservoir-like kinetics; higher % free than dietary | Strongest controlled evidence that single dermal contact can drive multi-day exposure and a higher unconjugated fraction vs ingestion |
| Liu & Martin 2019 | Controlled human trial + reconstructed epidermis comparison (BPA vs BPS) | Human: 6 recruited; 5 analyzed (P6 excluded). In vitro: n=3 tissues/dose | Human: native BPS thermal receipts (and BPA-d16 simulated receipt for comparison in same participants); In vitro: reconstructed human epidermis | Human: handle BPA-d16 receipt 5 min then BPS receipt 5 min; glove 2 h; wash; serum 2.5/5/7.5 h; urine 0–48 h. In vitro: 1.5 & 7.7 µg/cm² over 25 h | Urine/serum free + total BPS; (BPA-d16 compared to prior study); hand wipes; in vitro receiver/tissue | Hand wipes: mean 3.9 µg BPS vs 0.77 µg BPA-d16. Total BPS detected in 78% of urine samples; free BPS in 13%. No Serum BPS | Suggests lower percutaneous absorption of BPS vs BPA, but higher free fraction of BPS in urine implies potentially lower biotransformation efficiency | Human trial compares BPS with published BPA-d16 kinetics in same participants; background BPS in pre-urine complicates interpretation (not isotope-labeled) |
| **Sasso** *et al.* (**2020)** | Controlled human TK (dermal administration of labeled BPA) | 10 (6M, 4F) | **d6-BPA** applied to volar forearm (Hill Top chamber; occlusive) | **100 µg/kg** over **12 h**; vehicles: **0.3% CMC** or **95% ethanol**; blood + urine followed **3–6 days** | Serum & urine **total and free d6-BPA** | Total d6-BPA detectable in serum at **~1.4 h**; **Cmax total 3.26 nM**. Free d6-BPA detectable at **~2.8 h**; **Cmax free 0.272 nM**. Free + total serum plateaued ~**7–12 h** (during exposure). | Long apparent half-life; “skin-depot/flip-flop” kinetics; higher free:total vs oral | High-dose, **occlusive** regimen designed to characterize kinetics (may overestimate absorption vs typical receipt handling); useful for PBPK calibration and interpreting biomonitoring timing. |

**S-6. Summary of Occupational / Real-World In Vivo Biomonitoring Studies**

| Study | Occupational Setting | Sample Size (Exposed / Control) | Bisphenols Measured | Content | Study Design | Evidence Supporting Dermal Route | Major Findings |
| --- | --- | --- | --- | --- | --- | --- | --- |
| Heinälä *et al.* (2017) | Paint, thermal paper, composite, and tractor manufacturing workers | 48 / 121 reference population | BPA | Thermal paper manufacturing included BPA-containing coating material | Occupational biomonitoring with repeated timed urine sampling plus air monitoring | Strong supportive evidence; low air but high urinary BPA suggested skin exposure | Highest urinary BPA occurred in thermal paper and some paint tasks; coating machine workers and coating-material workers showed markedly elevated urinary BPA, with findings pointing to relevant dermal exposure |
| Hines *et al.* (2017a) | BPA manufacturing and BPA-use industries | 77 / 0 | BPA (total and free) | Not receipt-based | Repeated urinary biomonitoring over two workdays | Indirect; route not isolated | Urinary BPA in workers was far above general-population levels and increased across shifts/workdays |
| Hines *et al.* (2017b) | BPA manufacturing and BPA-use industries | 78 / 0 | BPA | Not receipt-based | Personal air, hand wipe, and surface wipe sampling over two workdays | Supportive evidence for dermal contribution | End-shift hand BPA was ~10-fold higher than pre-shift; production surfaces were highly contaminated; findings supported both inhalation and dermal exposure |
| Hines *et al.* (2018) | BPA manufacturing workers | 77 / 0 | BPA | Not receipt-based | Linked urine, air, and hand-wipe measures over two workdays | Strong; both dermal and inhalation supported | Urinary BPA correlated strongly with both air and hand BPA, but air appeared the more dominant route on average |
| Jang *et al.* (2022) | Receipt-handling volunteers | 24 / 0 | BPA, BPS | Conventional receipts contained BPA; BPA-free receipts contained BPS | Short-term human fingerprint transfer study | Strong for direct skin transfer | Measurable BPA/BPS transferred to fingertips within 20 s of handling receipts |
| Lee *et al.* (2018) | Female supermarket cashiers | 54 / 0 | BPA | BPA mean 9080 μg/g (~0.91% w/w) | Real-world glove intervention; pre-/post-shift | Strong; glove use suppressed post-shift rise | BPA roughly doubled post-shift without gloves, but not with gloves |
| Lv *et al.* (2017) | Simulated cashier receipt handling volunteers | 6 / 0 | BPA, TCS, 8-OHdG | Thermal receipts contained 23.5 mg BPA/g paper | Controlled real-world simulation with repeated urine sampling | Strong for dermal contribution | Urinary BPA rose about 3-fold during receipt handling and dermal route was estimated to contribute ~51.9–84.8% of urinary BPA; BPA correlated with urinary 8-OHdG |
| Ndaw *et al.* (2016) | Cashiers across multiple workplaces | 90 / 44 | BPA (free and total) | BPA in receipts: 0.93–1.81% w/w | Occupational urinary biomonitoring using 24-h and spot urine | Supportive; real-world receipt exposure, route not isolated | Total urinary BPA was significantly higher in cashiers than controls; free BPA did not differ significantly |
| Ndaw *et al.* (2018) | Hypermarket cashiers | 17 / 15 | BPS (free and total) | BPS in receipts: 1.2–1.4% w/w | Occupational urinary biomonitoring with pre-/post-shift and first-morning urine | Supportive; real-world receipt exposure, route not isolated | Total urinary BPS was significantly higher in cashiers than controls and higher post-shift than pre-shift |
| Porras *et al.* (2014) | Simulated receipt handling volunteers | 3 / 121 reference population | BPA | Thermal paper contained 0.9% BPA | Controlled receipt-handling simulation with repeated urine sampling | Limited; simulated dermal handling but weak biomonitoring response | Repeated receipt handling did not clearly increase urinary BPA above background/reference levels |
| Porras *et al.* (2024) | Sewage-pipe relining and floor-coating workers | 15 / 151 reference population | BPA, BPF | Not receipt-based | Occupational biomonitoring with urine, air, and hand-wipe sampling | Supportive for dermal contamination overall, but not for parent bisphenol uptake | No clear occupational increase in urinary BPA or BPF despite workplace contact with epoxy-related materials |
| Thayer *et al.* (2016) | Retail cashiers | 77 / 25 | BPA, BPS, BPSIP | BPA, BPS, or BPSIP; ~1–2% w/w | Pre-/post-shift biomonitoring with non-cashier comparison | Suggestive, not isolated; within-shift urinary BPA was nonsignificant (p=0.10); BPS increased; BPSIP detection frequency did not significantly increase within shift. | Receipt handling associated with higher urinary BPS and greater BPA/BPSIP exposure in relevant cashier groups |

**S-7. Summary of Dermal Contact Studies**

| Author (Year) | Study and Approach Type | Bisphenols | Matrix | Experimental Transfer Measured? | General Population Exposure | Occupational Exposure | Key Contribution |
| --- | --- | --- | --- | --- | --- | --- | --- |
| Adeyemi *et al.* (2020) | Receipt survey + dermal exposure estimation | BPA | Nigerian thermal receipts | No | Mean 0.20 μg/day | Mean 9.89 μg/day | Reported relatively lower BPA levels and lower estimated exposure than several earlier receipt studies. |
| Akçetin *et al.* (2025) | Receipt survey + deterministic/probabilistic dermal exposure and risk assessment | BPA, BPS, BPB, BPZ, BPF, BPP | Thermal receipts | No | BPA 0.000445 µg/kg/day; BPS 0.000039 µg/kg/day. | BPA 0.0223 µg/kg/day; BPS 0.00195 µg/kg/day. | Showed widespread bisphenol analog occurrence and notable occupational cumulative risk. |
| Biedermann *et al.* (2010) | Experimental paper-to-skin transfer study | BPA | Thermal printer paper/receipts | Yes | Not reported | Potential exposure up to 71 μg/day under repeated-contact worst-case assumptions | Provided direct evidence of BPA transfer from thermal paper to skin. |
| Fan *et al.* (2015) | Multi-paper survey + transfer/migration tests + dermal exposure estimation | BPA | Thermal receipts, currencies, and common paper products | Yes | 36.45 ng/day | 1.54 × 10⁻³–248.73 μg/day | Showed transfer to currencies/common papers, faster migration in synthetic sweat, and incomplete removal after handwashing. |
| Gallimberti *et al.* (2020) | Paper product survey + dermal exposure estimation | BPA | Thermal receipts and other paper products | No | 14.5 ng/day | 1070 ng/day | Quantified BPA across multiple paper-product types; values refer to aggregate paper handling, not receipt-only worst-case exposure. |
| Geens *et al.* (2012) | Receipt survey + dermal exposure estimation | BPA | Belgian thermal receipts | No | Median 445 ng/day | Not reported | Estimated meaningful general-population dermal exposure from Belgian receipts. |
| Han *et al.* (2024) | Multi-paper survey + dermal exposure estimation | BPS and chlorinated BPS derivatives (Cl₁–Cl₄-BPS) | Thermal paper and other paper products | No | Clx-BPS up to 0.546 ng/day; thermal-paper BPS mean 81.1 ng/day | Clx-BPS up to 16.4 ng/day; thermal-paper BPS mean 2430 ng/day | Expanded exposure assessment beyond BPA/BPS to chlorinated BPS derivatives. |
| Lee *et al.* (2021) | Receipt survey + dermal exposure estimation | BPA, BPS | Thermal receipt papers | No | BPA 0.08–602 ng/day; BPS 243–694 ng/day | BPA 6.1–45,123 ng/day; BPS 18,228–52,065 ng/day | Documented ongoing BPA-to-BPS substitution in Korean receipts and substantial occupational intake potential. |
| Liao & Kannan (2011a) | Paper product survey + dermal exposure estimation | BPA | Thermal receipts and other paper products | No | Median 17.5 ng/day; 5th–95th percentile 0.066–544 ng/day | Median 1303 ng/day; 5th–95th percentile 2.13–40,593 ng/day | Identified thermal receipts as the dominant paper-related dermal BPA source. |
| Liao & Kannan (2011b) | Currency survey + contact transfer experiment + dermal exposure estimation | BPA | Paper currencies | Yes | Mean 0.0001–2.10 ng/day across countries | Mean 0.0007–21.0 ng/day across countries | Showed receipt-to-currency transfer as a secondary BPA exposure source. |
| Liao *et al.* (2012) | Paper/currency survey + dermal exposure estimation | BPS | Thermal receipts, currencies, and other paper products | No | Median 4.18 ng/kg bw/day; 95th percentile 11.0 ng/kg bw/day | Median 312 ng/kg bw/day; 95th percentile 821 ng/kg bw/day | Established thermal receipts as the major paper-related dermal source of BPS. |
| Lu *et al.* (2013) | Receipt survey + probabilistic dermal exposure assessment | BPA | Supermarket receipts | No | Mean 0.69 μg/day; range 0.24–3.98 μg/day | Mean 40.4 μg/day; range 2–347 μg/day | Reported high BPA levels in supermarket receipts and notable cashier exposure potential. |
| Nasello *et al.* (2022) | Receipt study + dermal exposure estimation plus environmental migration testing | BPA | Thermal paper tickets | No | 1.6 ± 0.4 μg/day | 79.3 ± 19.5 μg/day | Linked high BPA levels in Argentine thermal paper to modeled dermal exposure; paper-to-soil and paper-to-water migration were also rapid. |
| Rocha *et al.* (2015) | Receipt survey + dermal exposure estimation | BPA, BPS | Brazilian thermal receipts | No | 5th percentile 0.44 μg/day; median 1.42 μg/day; 95th percentile 2.0 μg/day | 5th percentile 21.8 μg/day; median 71 μg/day; 95th percentile 101 μg/day | Reported high BPA/BPS in Brazilian receipts and substantial occupational exposure potential. |
| Russo *et al.* (2017) | Receipt survey + dermal exposure estimation | BPA, BPS | Italian thermal receipts | No | BPA 0.0625 μg/day; BPS 0.0244 μg/day | BPA 66.8 μg/day; BPS 15.6 μg/day | Showed widespread BPA/BPS co-occurrence; occupational values were based on a worst-case scenario. |
| Semerjian *et al.* (2023) | Receipt survey + dermal exposure estimation | BPA | Thermal receipts | No | 8.22 × 10⁻¹¹–0.000812 μg/kg bw/day | 7.89 × 10⁻⁹–0.0681 μg/kg bw/day | Found many receipts above the EU BPA content limit, though modeled dermal intakes remained below cited Tolerable Daily Intake (TDI) benchmarks. |
| Shin *et al.* (2026) | Multi-paper survey + dermal exposure assessment by business type | BPA and analogues, especially BPA and BPS | Thermal receipts and selected non-receipt papers | No | Thermal receipts mean 119 ng/day; other papers mean 1.82 ng/day | Large retail chains 4,410 ng/day; small local stores 11,700 ng/day; public service 9,930 ng/day | Showed strong business-type differences, with highest occupational exposure in small local stores. |
| Yang *et al.* (2019) | Multi-paper survey + dermal exposure estimation | BPA and multiple BPA alternatives | Thermal and nonthermal paper products | No | Total analytes: mean 1.77 μg/day; median 1.64 μg/day; worst case 4.24 μg/day | Not reported | Showed that focusing only on BPA/BPS underestimates dermal exposure because multiple alternative developers contribute. |

**S-8. Summary of Dermal Contact from Textiles, Wearables, and Consumer Materials Studies**

| Study | Matrix / Exposure Source | Population Modeled | Bisphenols Assessed | Concentration Range (Key Findings) | Exposure Model Type | Estimated Dermal Dose (Magnitude) | Key Findings |
| --- | --- | --- | --- | --- | --- | --- | --- |
| Blignaut *et al.* (2026) | Sanitary pads and panty liners (top sheet) | Adult women | BPA, BPF, BPS, BPAP | Pads: BPA 11.85–77.32 ng/g, BPF 20.07–27.26 ng/g, BPS 0.381–3.072 ng/g. Liners: BPA 18.18–56.08 ng/g, BPS 0.417 ng/g, BPAP 32.67 ng/g | Deterministic dermal model with normal- and high-absorption scenarios | Bisphenol Dermal Exposure Dose (DED): 19.94 ng/kg/day in pads and 12.89 ng/kg/day in liners (normal-skin scenario) | Bisphenols were major contributors in pads; the paper highlights greater concern for vulvar exposure scenarios |
| Chabowska *et al.* (2023) | Disposable baby diapers (absorbent core and supporting wings) | Newborns and infants | BPA, BPF, BPS, BPZ (+ BADGE/BFDGE derivatives) | BPA: 5.0–520 ng/g; BADGE·2HCl: 6.8–530 ng/g | Deterministic infant dermal exposure model with 8.6%, 20%, and 50% absorption scenarios | The highest reported 95th-percentile modeled dose was 412 ng/kg body weight/day for BADGE·2HCl, compared with 172 ng/kg body weight/day for BPA in the reported size-6 diaper scenario | BPA was the most frequent analyte; exposure estimates were much higher than those reported for many textile studies |
| Ding *et al.* (2022) | School supplies (paper, plastic, fabric, rubber) | General population and occupational users/students | BPA, BPF, BPS (+ 15 BPs total) | Σ15 BPs median: 817 ng/g; paper median: 1347 ng/g; BPA median: 161 ng/g; max BPA: 212,600 ng/g | Deterministic dermal Estimated Daily Intake (EDI) / Body Weight (BW)-EDI model | BW-EDI median: 2.61 ng/kg/day (general) and 7.21 ng/kg/day (occupational); high-end students up to 1527 ng/kg/day | Paper was the dominant source; high-end handling scenarios were much higher than average |
| Freire *et al.* (2019) | Socks for infants and young children | Infants/children (1–48 months) | BPA | BPA: <0.70–3736 ng/g; median 20.5 ng/g | Deterministic dermal estimate - ECHA dermal model | Mean BPA dose 19.6 pg/kg/day; highest age-group median 17.6 pg/kg/day | BPA widely detected in socks; higher levels in store 1 socks |
| Gao & Kannan (2020) | Feminine hygiene products (pads, panty liners, tampons, wipes, creams, sprays, powders) | Adult women | BPF, BPA, BPS (+ other bisphenols screened) | Pads Σbisphenols median 5.59 ng/g; panty liners 21.1 ng/g; tampons 5.56 ng/g; BPA in pads up to 55.9 ng/g and panty liners up to 157 ng/g | Deterministic dermal/mucosal exposure model with multiple absorption scenarios | Median bisphenol dose: 0.039 ng/kg bw/day (pads), 0.097 (panty liners), 0.064 (tampons) | Panty liners had the highest median bisphenol burden; exposure depends strongly on assumed absorption through vulvar/vaginal tissues |
| Herrero *et al.* (2023) | New clothes for pregnant women, newborns, and toddlers | Pregnant women, newborns, toddlers | BPA, BPB, BPF, BPS | BPA: 0.69–5872 ng/g; BPB: <LOD–44.1 ng/g; BPF: <LOD–11,333 ng/g; BPS: <LOD–981 ng/g | Deterministic dermal exposure/risk characterization - ECHA dermal model | BPA total dermal exposure: 4.94×10⁻10 to 1.24×10⁻9 mg/kg/day | BPA detected in all samples; polyester showed the highest median levels |
| Jurikova *et al.* (2024) | T-shirts and socks from conventional and recycled textiles | Adults | BPA, BPS, BPF, BPB | BPA: <0.050–625 ng/g; BPS: 0.277–2474 ng/g; BPF up to 57.3 ng/g; BPB not detected | Deterministic dermal model for dry and sweat-wet textiles - ECHA dermal model | Dry: BPA up to 209 pg/kg/day; Wet: BPA up to 114 ng/kg/day | The highest dry-textile modeled BPA dose was 209 pg/kg body weight/day (0.209 ng/kg/day), slightly above the numerical value of the EFSA 2023 BPA TDI (0.2 ng/kg/day). Sweat-wet estimates were substantially higher under different measured migration and assumed penetration inputs. |
| Li & Kannan (2018) | Pantyhose | Adult women | BPA, BPS, BPF | BPS: <0.3–2,190,000 ng/g; BPA: <1.3–504 ng/g | Deterministic dermal model | Dermal doses varied by garment length; BPS up to 45,900 pg/kg/day | BPS was dominant and found in 100% of samples; dissolution extraction yielded much higher values than ultrasonic extraction |
| Wang *et al.* (2019) | Daily clothes (used and new); sweat and laundering scenarios | Adults and children | BPA, BPS (+ analogues screened) | Used clothes BPA: <3.30–471 ng/g; median 34.2 ng/g. New clothes BPA: max 1823 ng/g; median 17.7 ng/g | Deterministic dermal model with laundering and sweat-leaching experiments | BPA up to 52.1 ng/kg bw/day in children from highly polluted sweaty clothes | Used clothes remained an important BPA source; laundering caused cross-contamination |
| Xue *et al.* (2017) | Textiles and infant clothing, including socks, diapers, blankets, clothes | Newborns and infants (<1 year) | BPA, BPS (+ BADGEs/NOGEs also analyzed) | BPA: <2.21–13,300 ng/g; BPS: <0.74–394 ng/g | Deterministic infant dermal exposure model | Mean BPA dose 201–248 pg/kg bw/day; maximum BPA dose 7280 pg/kg bw/day | Socks had the highest BPA levels and dominated infant textile exposure |
| Zhu *et al.* (2020) | Indoor dust | Infants, toddlers, children, teenagers, adults | BPA, BPF, BPS (+ 8 BPs total) | Σ8 BPs: 8.80–37,400 ng/g; BPA median 214 ng/g; BPF median 32.9 ng/g; BPS median 5.64 ng/g | Deterministic ingestion + dermal absorption model | Dermal Σ8 BPs median: 0.020–0.088 ng/kg bw/day, age-dependent | Dermal exposure was smaller than ingestion, but infants had the highest combined exposure |

**S-9. Summary of Dermal Modeling and Probabilistic Exposure Estimation Studies**

| Study | Modeling approach | Bisphenols | Exposure scenario | Key modeling features | Major findings |
| --- | --- | --- | --- | --- | --- |
| Akçetin *et al.* 2025 | Deterministic + probabilistic risk assessment (thermal paper) | BPA, BPS, BPB, BPZ, BPF, BPP (and “PCs” framing) | Thermal receipt papers (public vs occupational) | Explicitly reports deterministic EDI/ hazard quotient (HQ) / Hazard Index (HI); includes probabilistic calculation section; parameter ranges for k, HF, HT etc | Notes modeled exposures can exceed EFSA’s (new) TDI in some cases ; reports wide HQ/HI ranges including HI>1 scenarios |
| Bernier & Vandenberg (2017) | Deterministic exposure estimation using observed handling behavior + literature parameters | BPA (and discusses alternatives qualitatively) | Thermal paper handling (behavioral observations in cafeteria setting) | Intake equation uses transfer coefficient k, absorption factor AF, time/area assumptions; compares to EFSA assumptions | Conservative modeled intake ~51.1 ng/kg/day, ~EFSA’s 59 ng/kg/day; less conservative parameter choices imply higher intakes |
| Blignaut *et al.* 2026 | Deterministic DED + scenario analysis | BPA plus analogues (BPF, BPS, etc.) within a broader EDC set | Sanitary pads & pantyliners (top sheet; dermal contact) | DED equation; assumes N≈6/day, BW≈75 kg; uses multiple absorption scenarios because vulvar-specific rates unavailable | Reports that, under “normal skin” assumptions, BPA DED exceeds EFSA TDI 0.2 ng/kg/day by ~60× (pads) and ~32× (liners); also reports exceedances for some analogues |
| Fu *et al.* (2016) | Stochastic integrated risk assessment: exposure distributions + PBPK + Hill models | BPA | Multiple routes incl. food and nonfood sources (explicitly includes thermal paper) | Uses lognormal exposure distributions; Monte Carlo 10,000 iterations; PBPK estimates urinary BPA; examines outcomes with/without skin absorption | Risk characterization suggests similar order-of-magnitude conclusions with or without skin absorption in their framework |
| Hu *et al.* 2023 | Advanced dermal PBPK (parallel-layered skin) + Monte Carlo (MC) uncertainty + sensitivity analysis | BPA, BPS, BPF, BPAF | Thermal paper (BPS; applied to BPA conservatively) + PCPs (BPF/BPAF) | Replaces single skin compartment with Stratum Corneum (SC)+ Viable Epidermis (VE)+ follicle shunt + skin-surface depot; includes Fickian diffusion + desquamation; MC n=10,000 | Reports/uses bioaccessibility estimates (e.g., BPS via TP) and applies them in risk-conservative ways (e.g., applying BPS bioaccessibility to BPA) |
| Karrer *et al.* (2018) | PBPK for BPA, BPS, BPF, BPAF with updated metabolic parameters | BPA, BPS, BPF, BPAF | Peroral + dermal (thermal paper + PCP use scenarios) | Simulates dermal via TP touch + PCP use twice/day; uses dermal absorption fractions 20% (TP) and 60% (PCPs); dermal half-lives 6 h (TP) and 10 min (PCPs) | Establishes route-specific scenario inputs for bisphenol analog PBPK comparison |
| Karrer *et al.* (2019) | Probabilistic aggregate exposure + cumulative risk (cumulative risk assessment (MCRA) + external probabilistic models) | BPA, BPS, BPF, BPAF (TP focus mainly BPA/BPS occurrence) | Europeans; dietary + dermal PCP + thermal paper + dust; “before vs after restrictions” | Uses MCRA; PCP exposure via consumer product exposure (PACEM); TP/dust via self-developed probabilistic models; 10,000 MC runs per stratum | Quantifies aggregate/cumulative internal exposures to BPA-equivalents under legislative scenarios |
| Knudsen *et al.* (2015) | Parallelogram method–based dermal exposure assessment | TBBPA (brominated bisphenol) | Dermal contact scenario relevant to dust-related exposures | Applies parallelogram approach; notes ~±3-fold uncertainty; estimates ~6% bioavailable dermally applied TBBPA | Provides a plausible human bioavailability estimate for dermal TBBPA exposure |
| Mielke *et al.* (2011) | Human PBPK updated to include dermal absorption | BPA | Thermal paper/receipt-like dermal exposure; aggregate context | Dermal absorption framed with literature absorption range (10–46%, possibly ~60%); includes skin metabolism assumption | For a modeled dermal intake example, dermal exposure could account for ~11–64% of the highest urinary total BPA reported in biomonitoring datasets |
| Wiśniowska *et al.* (2023) | Mechanistic dermal absorption , Multi-Phase Multi-Layer Mechanistic Dermal Absorption (MPML MechDermA, Simcyp) + PBPK | BPA | Thermal paper hand-contact case study | Uses MPML MechDermA + PBPK; toxicokinetic study for refinement; dose reconstruction from cumulative urinary excretion; compares to simpler model; sensitivity analysis | Predicts stratum corneum depot/reservoir behavior and discusses implications for linking external-to-internal exposure |

**Note**

Unless stated otherwise, percentages use applied dose as denominator. Receptor recovery, skin retention, dermal delivery, urinary recovery, and systemic bioavailability are separately identified. NR denotes not reported in the checked source; source-pending denotes a value not independently verified. Neither is zero.
